# Transposon-Mediated Horizontal Transfer of the Host-Specific Virulence Protein ToxA between Three Fungal Wheat Pathogens

**DOI:** 10.1128/mBio.01515-19

**Published:** 2019-09-10

**Authors:** Megan C. McDonald, Adam P. Taranto, Erin Hill, Benjamin Schwessinger, Zhaohui Liu, Steven Simpfendorfer, Andrew Milgate, Peter S. Solomon

**Affiliations:** a Division of Plant Sciences, Research School of Biology, The Australian National University, Canberra, Australia; b Department of Plant Pathology, North Dakota State University, Fargo, North Dakota, USA; c NSW Department of Primary Industries, Tamworth Agricultural Institute, Tamworth, NSW, Australia; d NSW Department of Primary Industries, Wagga Wagga Agricultural Institute, Wagga Wagga, NSW, Australia; Universidad de Córdoba

**Keywords:** horizontal transfer, transposon, fungal wheat pathogen, adaptive evolution, ToxA, fungal pathogen, wheat pathogen

## Abstract

Most known examples of horizontal gene transfer (HGT) between eukaryotes are ancient. These events are identified primarily using phylogenetic methods on coding regions alone. Only rarely are there examples of HGT where noncoding DNA is also reported. The gene encoding the wheat virulence protein ToxA and the surrounding 14 kb is one of these rare examples. *ToxA* has been horizontally transferred between three fungal wheat pathogens (Parastagonospora nodorum, Pyrenophora tritici-repentis, and Bipolaris sorokiniana) as part of a conserved ∼14 kb element which contains coding and noncoding regions. Here we used long-read sequencing to define the extent of HGT between these three fungal species. Construction of near-chromosomal-level assemblies enabled identification of terminal inverted repeats on either end of the 14 kb region, typical of a type II DNA transposon. This is the first description of *ToxA* with complete transposon features, which we call ToxhAT. In all three species, ToxhAT resides in a large (140-to-250 kb) transposon-rich genomic island which is absent in isolates that do not carry the gene (annotated here as *toxa^−^*). We demonstrate that the horizontal transfer of ToxhAT between P. tritici-repentis and P. nodorum occurred as part of a large (∼80 kb) HGT which is now undergoing extensive decay. In B. sorokiniana, in contrast, ToxhAT and its resident genomic island are mobile within the genome. Together, these data provide insight into the noncoding regions that facilitate HGT between eukaryotes and into the genomic processes which mask the extent of HGT between these species.

## Abstract (precis)

This work dissects the tripartite horizontal transfer *of ToxA*, a gene that has a direct negative impact on global wheat yields. Defining the extent of horizontally transferred DNA is important because it can provide clues to the mechanisms that facilitate HGT. Our analysis of *ToxA* and its surrounding 14 kb suggests that this gene was horizontally transferred in two independent events, with one event likely facilitated by a type II DNA transposon. These horizontal transfer events are now in various processes of decay in each species due to the repeated insertion of new transposons and subsequent rounds of targeted mutation by a fungal genome defense mechanism known as repeat induced point mutation. This work highlights the role that HGT plays in the evolution of host adaptation in eukaryotic pathogens. It also increases the growing body of evidence indicating that transposons facilitate adaptive HGT events between fungi present in similar environments and hosts.

## INTRODUCTION

Horizontal gene transfer (HGT) is a mechanism whereby DNA from unrelated organisms is transferred between the organisms in a non-Mendelian fashion (1). In proteobacteria, HGT is thought to have occurred in over 75% of all protein families, making HGT one of the most important tools facilitating adaptation to stressful environments (2, 3). This propensity to share DNA between species has been attributed to many human health issues, such as the rapid rise and spread of antibiotic resistance in hospitals (4, 5). In eukaryotes, HGT was once thought to be a rare event and therefore not an important contributor to environmental adaptation. However, numerous studies have now shown that HGT between eukaryotes plays a very important role in adaptation, especially in the case of microbes that colonize a common host (6–8).

Among eukaryotic microbes, fungi are often used for kingdom-wide studies of adaptation, due to their relatively small genome size, importance in human and plant disease, and applications in food and biotechnology (6, 8, 9). Domesticated fungi, particularly those used in food production, are now being used as model organisms to understand the genetic basis of adaptation (10–12). On an evolutionary time scale, these organisms have been subjected to a short but intense period of selection, which has had dramatic effects on their preferred carbon and nitrogen sources, secondary metabolite production, and many other physiological traits (12, 13). One emerging theme from these studies is that organisms which are common contaminants of the food-making process are often donors of the genes that provide fitness advantages in these specialized environments. The reported HGT events are extensive and involve tens of thousands of bases of DNA, which remain over 90% identical between very distantly related species (10, 11). These HGTs contain both coding and noncoding regions which are stably integrated into the core nuclear genomes of the recipient species (10, 11). While the original reports suggested that these regions were important for adaptation to the domestic environment, the fitness advantage conferred by these genes had to be demonstrated in follow-up studies performed with knockout strains (13, 14).

Rapid adaptation via HGT is not restricted to domesticated species, but there exist very few described instances where the horizontally transferred DNA is integrated into the core nuclear genome and remains highly identical outside coding regions. One standout example is the virulence gene *ToxA* and the surrounding 11 to 12 kb, which to date has been reported in three fungal wheat pathogens: Parastagonospora nodorum, Pyrenophora tritici-repentis, and Biopolaris sorokiniana (13–16). While all three species belong to the same fungal order, the Pleosporales, they are distant relatives, with several million years separating their speciation (15, 16). Similar to the domesticated fungi discussed above, this HGT event is hypothesized to be extremely recent, as the average pairwise nucleotide identity across this 12 kb region remains greater than 92% (17). The *ToxA* gene itself remains identical between P. tritici-repentis and B. sorokiniana and only three nucleotides different from between B. sorokiniana and P. nodorum (17). The fitness advantage that *ToxA* confers has been demonstrated experimentally, whereby the presence of *ToxA* in a fungal isolate leads to faster development of necrotic lesions on wheat leaves (17, 18). This virulence function is genotype specific, as *ToxA* causes necrosis only on wheat lines that carry the susceptibility gene called *Tsn1* (19–21). In the absence of *Tsn1*, all three fungal species can still infect wheat due to the presence of other virulence genes (17, 19, 20).

Though *ToxA* confers a strong fitness advantage, this HGT event is not a fixed insertion and persists in all three pathogen populations as a presence/absence polymorphism (17, 21–23). The size of this presence/absence polymorphism has yet to be fully characterized. The frequency of *ToxA* in different field populations around the world also varies dramatically, ranging from 6% to 97% in different pathogen field populations (22). The selective forces that increase the frequency of *ToxA* in some fungal populations and decrease it in others remain unknown. Results of studies that examined whether there was a positive correlation between the frequency of *ToxA* in fields planted with *Tsn1* (susceptible) wheat cultivars were inconclusive (24). For ease of reading, here we use the notation *ToxA^+^* for isolates that contain the gene and *toxa^−^* for isolates that do not carry the gene.

Despite detailed knowledge on the molecular function of *ToxA* and its prevalence in fungal pathogen populations throughout the world, we still do not know the origins of this important virulence gene or the mechanisms that facilitated its transfer and stable integration into the genomes of these three pathogen species. There is clear evidence that *ToxA* is embedded in an AT-rich, repeat-dense region of the genome in all three species. AT richness in these portions of the genome is driven by a fungus-specific genome defense process known as the repeat induced point mutation (RIP) (25). RIP targets repeated sequences as small as 155 bp, mutating C:G to T:A, which introduces early stop codons in repeated DNA sequences (25–28). This mechanism is hypothesized to have evolved in some phyla of fungi to stop the spread of transposons or other self-copying elements within their genomes (26).

*ToxA* and its highly conserved flanking DNA provide a unique opportunity to dissect the integration of horizontally transferred DNA into the nuclear genomes of three fungal pathogens. To define the location and extent of each HGT event, we used long-read DNA sequencing to generate near-complete genome assemblies for several representatives from two of the three species, in addition to several other published assemblies (29, 30). We performed extensive *de novo* annotation of the repeat families in all three fungal species and manually annotated the region surrounding the *ToxA* gene. These assemblies and repeat annotations resolve the genomic context in which the virulence gene is located and provide insights into potential mechanisms of HGT as well as into the history of horizontal transfer events.

## RESULTS

### Long-read sequencing reveals a conserved type II DNA transposon.

The genomic location of *ToxA* has been best described in P. tritici-repentis, where two long-read assemblies place this gene in the middle of chromosome 06 (supercontig1.4) (23, 31). Several long-read assemblies have also been generated for *ToxA^+^* P. nodorum isolates Sn4 and Sn2000, where *ToxA* is found on chromosome 08 in both isolates (29). In addition to these publicly available assemblies, we sequenced *ToxA^+^* isolates P. nodorum SN15 and B. sorokiniana CS10 (original isolate name, BRIP10943) with seven PacBio SMRT cells each, resulting in approximately 500,000 reads with average read lengths of 10.6 kb and 9.4 kb, respectively. In addition to the two SMRT assemblies, we resequenced an additional four isolates with the Oxford Nanopore MinION. This included two *toxa^−^* isolates, P. nodorum isolate Sn79-1087 and B. sorokiniana isolate CS27 (original isolate name, BRIP27492a), as well as two additional *ToxA^+^* B. sorokiniana isolates, WAI2406 and WAI2411. All isolates were *de novo* assembled using long-read data only. Short-read Illumina data were used to “polish” the Nanopore *de novo* assemblies of CS27 and Sn79-1087. A complete list of all isolates used in this study and assembly quality indicators is provided in Table 1. Genome assembly accession numbers and additional information about the isolates are provided at https://github.com/megancamilla/Transposon-Mediated-transfer-of-ToxA/tree/master/S1_GenomeStats. B. sorokiniana chromosomes were ordered from largest to smallest and named based on the PacBio assembly of isolate CS10. Our P. nodorum contigs were named based on synteny alignments to the assemblies published recently by Richards et al. (29).

**TABLE 1:** Summary of genome assembly statistics for each isolate assembled in this study

Species and isolate	Raw data	ToxA genotype	Assembly size (Mb)^*a*^	No. of contigs^*b*^	No. of contigs with both telomeres	No. of contigs with 1 telomere	BUSCO score^*c*^
B. sorokiniana							
CS10	PacBio	ToxA^+^	36.9	22	14	3	98.9
CS27	Nanopore + Illumina	*toxa*^−^	35.2	19	NA^*d*^	NA	98.9
WAI2406	Nanopore	ToxA^+^	36.9	21	NA	NA	72.4
WAI2411	Nanopore	ToxA^+^	36.2	21	NA	NA	69.8

P. nodorum							
SN15	PacBio	ToxA^+^	37.3	26	18	6	99.1
Sn79-1087	Nanopore + Illumina	*toxa*^−^	34.7	23	13	8	92.4

a Size in millions of base pairs.

b Nuclear contigs only.

c Percentage of 1,313 ascomycete single-copy orthologs found in assembly.

d NA, not assessed.

Assembly quality was assessed using the Benchmarking Universal Single-Copy Orthologs (BUSCO) tool, which identifies fragmented, duplicated, and missing genes from *de novo* assemblies (32, 33). The score reported in Table 1 is the percentage of complete genes found in a set of 1,313 BUSCO genes from the Ascomycota odb9 data set. The number of complete genes is used as a proxy to estimate total-genome completeness (32). The assembly completeness scores were much lower for Nanopore-only assemblies in isolates WAI2406 and WAI2411, where no short-read data were available for genome correction. For isolates CS27 and Sn79-1087, available short-read data allowed correction of the assemblies, such that the proportion of complete BUSCO genes found exceeded 90%. Both PacBio assemblies, without short-read data, generated a BUSCO score above 98% (Table 1). For both the CS10 and SN15 PacBio assemblies, the 6-bp telomeric repeat (TAACCC) was found on the ends of several contigs (summarized in Table 1). We could not identify telomeric repeats in the Nanopore assemblies for the B. sorokiniana isolates. However, we were able to identify many contigs with telomeric repeats for the assembly of P. nodorum isolate Sn79-1087 (Table 1).

**FIG 1:**
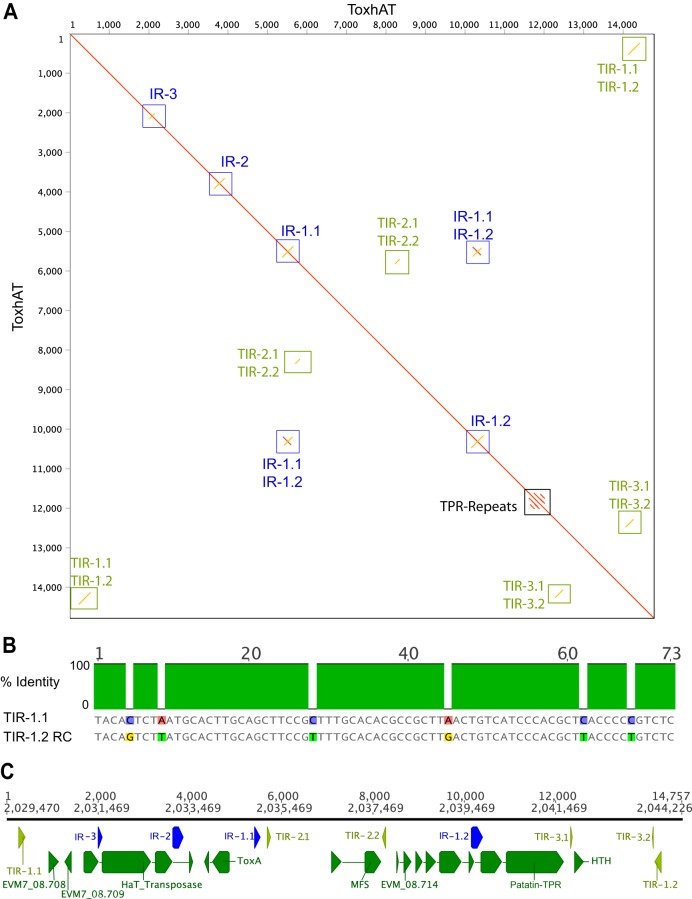
Characterization of ToxhAT in B. sorokiniana isolate CS10. (A) Self-alignment of ToxhAT drawn as a dot plot. The red line down the center indicates a 1-to-1 alignment; yellow lines show inverse alignments. Terminal inverted repeats (TIRs) and inverted repeats (IRs) are boxed. The TPRs are short tandem repeats found in the gene with the Patatin domain. (B) Alignment of 74-bp TIR1.1 and the reverse complement (RC) of TIR1.2. Gray bases indicates aligned positions that are identical between the two sequences. (C) Manual annotation of coding regions within ToxhAT showing each open reading frame (green), inverted repeat (blue), and TIR (light green).

We have previously reported the presence of >92% identical 12 kb region shared between all three species (17). This aligns with the original publication from Friesen et al. (20), in which an 11 kb element was reported to be conserved between P. tritici-repentis and P. nodorum. In both P. nodorum SN15 and B. sorokiniana CS10, the chromosomes that contain *ToxA* were assembled completely, with telomeric repeats on both ends. A self-alignment of this region in B. sorokiniana CS10 revealed intact, terminal inverted repeats (TIRs) separated by 14.3 kb (Fig. 1A). TIRs are structural features of type II DNA transposons of the order “TIR,” which are required for excision by transposases (34). These TIRs were not identified in previous studies (17, 20), which we explore further below. The aligned TIRs consist of 74 bp and are ∼92% identical (Fig. 1B). Here, we refer to *ToxA* and the accompanying noncoding and coding DNA enclosed within these TIRs as “ToxhAT.*”* This name reflects the historical association of *ToxA* with the neighboring type II hAT-like transposase gene (20).

We annotated the coding regions within ToxhAT in B. sorokiniana isolate CS10 with both long-read and short-read RNA sequencing (RNA-seq) data (see Fig. S1 in the supplemental material). This annotation plus the self-aligned sequence revealed eight genes, three inverted repeats (IRs), and two additional internal TIRs (Fig. 1C). Three annotated genes, *CS10_08.708*, *CS10_08.709*, and *CS10_08.714*, contain no known protein-coding domains. Excluding *ToxA*, the remaining four genes had conserved domains, as identified by NCBI's conserved domain database search tool (Fig. 1C). One gene contained a major facilitator superfamily (MFS) domain (NCBI accession no. cl28910), which in yeast was shown to be a proton-coupled transporter of dipeptides and tripeptides (35). The largest coding DNA sequence (CDS) within ToxhAT contained two known protein domains, with a Patatin domain at the N terminus (accession no. cd07216) followed by tetratricopeptide repeats (TPR; pfam13424) at the C terminus. In fungi, proteins that contain these domains are recognized as members of the NOD-like receptor (NLR) family (36). Only a limited number of these proteins have been functionally studied in fungi, but they are considered to be broadly involved in self-recognition and immunity (36, 37). The fourth gene was flanked by its own set of TIRs and contained a helix-turn-helix (HTH) DNA binding domain (accession no. cl04999). This structure indicated that this CDS likely represents a nested type II transposase within ToxhAT (Fig. 1C). This indicates that ToxhAT is a composite of at least two DNA transposons. Fragments of the eight open reading frames (ORFs) are also found in the other two species, P. nodorum and P. tritici-repentis (Fig. S2); however, the 3′ end of ToxhAT is invaded by unique sequences in these two species (Fig. S2).

10.1128/mBio.01515-19.1 FIG S1 Alignment of Illumina and MinION RNA sequencing reads from *in vitro* cultures of B. sorokiniana isolate CS10 against its genomic sequence. Three panels are shown with the aligned reads represented as gray bars. Small polymorphisms are shown as colored vertical lines within the gray reads. Indels are indicated by small purple lines with the gray reads. The top panel shows the combined Illumina reads from all seven *in vitro* growth conditions. The middle panel shows reads from the Fries 3 media alone, where the highest expression of *ToxA* was observed. The bottom panel shows the MinION reads also from Fries 3 media. Genes annotated by the pipeline described in Materials and Methods are shown in the blue boxes at the bottom of the plot. Two additional coding regions were manually added on the basis of predicted open-reading frames, namely, the MFS transporter gene (yellow box in schematic) and the Tc1 transposase open reading frame (blue box in schematic). The terminal inverted repeats (TIRs) and other coding regions of ToxhAT are indicated in the schematic at the bottom of the figure. Download FIG S1, PDF file, 0.1 MB. Copyright © 2019 McDonald et al. 2019 McDonald et al. This content is distributed under the terms of the Creative Commons Attribution 4.0 International license.

10.1128/mBio.01515-19.2 FIG S2 Annotated ToxhATs from TIR to TIR extracted from the genome assembly of each species. The AT richness across each sequence is shown in the line plot underneath the annotations, with AT richness shown in green and GC richness shown in blue. (A) B. sorokiniana ToxhAT track (1) showing annotated genes and other features of ToxhAT. (B) P. tritici-repentis ToxhAT. Track (1) shows genes and other features from CS10 that are >90% identical in P. tritici-repentis 1C-BFP; track (2) shows annotated genes from P. tritici-repentis 1C-BFP; track (3) shows repeat annotations from REPET. (C) P. nodorum SN15 ToxhAT. Track (1) shows genes and other features from CS10 that are >90% identical in P. nodorum (yellow indicates pseudogene of CS10 FabD/phospholipase-like protein); track (2) shows annotated genes from annotated genes from P. nodorum SN15; track (3) shows repeat annotations from REPET. (D and E) Alignment of the (D) 5′ and (E) 3′ terminal inverted repeats (TIRs) of ToxhAT between all three species. This alignment includes an additional 300 bp outside ToxhAT to show the sequence shared between P. nodorum and P. tritici-repentis. Gray rectangles represent areas of the alignment that are identical between the three isolates. Colored vertical lines indicate base pair positions where there are differences between two isolates. Purple lines indicate C’s, green lines indicate T’s, yellow lines indicate G’s, and red lines indicate A’s. Thin black lines show indels in the alignment. The light-green annotation box below the gray line in B. sorokiniana shows the location of the 74-bp TIR shown in the alignment in Fig. 1C. This alignment highlights the many mutations of C (purple) to T (green) and of G (yellow) to A (red) which are typical of repeat induced point mutations (RIP). Download FIG S2, PDF file, 1.3 MB. Copyright © 2019 McDonald et al. 2019 McDonald et al. This content is distributed under the terms of the Creative Commons Attribution 4.0 International license.

**FIG 2:**
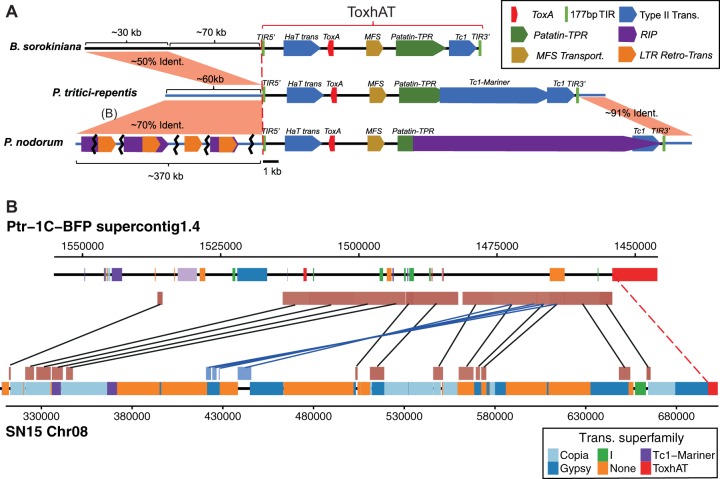
(A) Overview of the ToxhAT in all three pathogen species. All features drawn to the right of the red dashed line are drawn to scale as indicated by the black scale bar at the bottom. Features to the left of the red dashed line are not drawn to scale; relative sizes are indicated with brackets. The opaque red rectangles drawn between the chromosomes outside the TIRs show regions of synteny as indicated by whole-chromosome alignment. The approximate percentage of nucleotide identity is indicated within the red shading. “(B)” in part A indicates the region shared between P. nodorum and P. tritici-repentis, which is drawn in part. (B) Closeup view of whole-chromosome alignment between P. nodorum and P. tritici-repentis. Chromosomes are drawn as thick black lines with positions of annotated transposons shown in colored blocks. Transposons are classified into superfamiles as indicated by the legend. The additional opaque red/blue boxes appearing above or below the chromosomes represent nucleotide regions that are >70% identical and were identified by whole-chromosome alignment with LASTZ. Black lines connect syntentic blocks aligned in the same direction, while blue lines connect inverted syntenic blocks. Trans, transposon.

Using B. sorokiniana as a guide, we were able to identify remnants of the ToxhAT TIRs in both P. tritici-repentis and P. nodorum. The 5′ TIR in P. tritici-repentis remains largely intact, whereas the 3′ TIR is enriched in the C-to-T and G-to-A transitions characteristic of RIP (Fig. S2). In P. nodorum, both the 5′ and 3′ TIR are enriched in RIP mutations, which, without prior knowledge of the TIR location in B. sorokiniana, would be impossible to identify. There were additional unique sequence insertions inside ToxhAT in P. tritici-repentis and P. nodorum (Fig. 2; see also Fig. S2). Manual annotation of this unique sequence showed P. tritici-repentis 1C-BFP has a 3′ insertion of a type II DNA transposon sequence, a Tc1-mariner-like sequence, which interrupts ToxhAT, separating the 5′ TIR from the 3′ TIR by ∼20.2 kb. In P. nodorum SN15, ToxhAT is interrupted by a different transposon which resembles a type I long-terminal-repeat (LTR) retrotransposon. The exact identity of this transposon was difficult to determine due to the presence of extensive RIP-like mutations in this sequence. This insertion separates the ToxhAT TIRs in P. nodorum by ∼25.6 kb (Fig. S2). Despite these additional insertions, the TIRs identified in B. sorokiniana were found to be present, though heavily mutated, in the other two species, indicating a common evolutionary origin for ToxhAT.

### ToxhAT was transferred in two independent HGT events.

Whole-chromosome alignments (WCA) between the ToxhAT-containing chromosomes of P. nodorum and P. tritici-repentis revealed DNA with >70% sequence identity beyond the boundaries set by ToxhAT TIRs (Fig. 2A; see also Fig. S2). Pairwise alignments of these regions revealed that almost all polymorphisms were RIP-like (Fig. S3). Excluding the RIP-like mutations, the sequence identity between the two species is nearly 100%. Ten genes annotated in P. tritici-repentis 1C-BFP, PTRG-04890 to PTRG-04909, are all present in P. nodorum SN15 upstream of the 5′ ToxhAT TIR. In P. nodorum, however, each of these was a pseudogene due to RIP and therefore has not been annotated in any assembly (Fig. S3) (21, 29). Furthermore, 5 of these 10 genes, PTRG-04891 to PTRG-04895, are duplicated and found in inverse orientation within the P. nodorum SN15 assembly (Fig. 2B, blue boxes). In P. tritici-repentis 1C-BFP, these 10 genes are on a contiguous piece of DNA that extends 61.2 kb upstream of the ToxhAT 5′ TIR. The total length of nearly identical sequence shared between these two species is ∼80 kb, which includes 61.2 kb upstream and 1.7 kb downstream of ToxhAT. In P. nodorum SN15, the 61.2 kb shared with P. tritici-repentis 1C-BFP is present but highly fragmented across chromosome 08, spanning nearly ∼370 kb (Fig. 2B). These data demonstrate that a specific HGT event occurred between P. tritici-repentis and P. nodorum that included ToxhAT and a large segment of surrounding DNA.

10.1128/mBio.01515-19.3 FIG S3 Representative alignments of the HGT region shared between P. tritici-repentis and P. nodorum outside ToxhAT. These alignments span five genes originally annotated in P. tritici-repentis 1C-BFP. These genes are shown in red boxes between the two aligned sequences and have the names in the form PTRG_04###. These alignments show that the point mutations between these two species in this region are nearly exclusively RIP-like. Gray indicates identical sequences; vertical yellow or purple lines indicate G or C; vertical red or green lines indicate A and T. The green bars in the “Identity” plot show alignment positions where the nucleotides match. (A) Representative alignment of HGT outside ToxhAT between P. tritici-repentis and P. nodorum, spanning the annotated genes PTRG_04908 and PTRG_04909. The pairwise nucleotide identity for this alignment is 75.8%. (B) Representative alignment of HGT outside ToxhAT between P. tritici-repentis and P. nodorum spanning the annotated genes PTRG_04893 and PTRG_04894. The pairwise nucleotide identity for the alignment shown is 76.8%. (C) Representative alignment of HGT outside ToxhAT between P. tritici-repentis and P. nodorum spanning the annotated gene PTRG_04890. The pairwise nucleotide identity for the alignment shown is 78.5%. Download FIG S3, PDF file, 2.3 MB. Copyright © 2019 McDonald et al. 2019 McDonald et al. This content is distributed under the terms of the Creative Commons Attribution 4.0 International license.

WCA between P. tritici-repentis and B. sorokiniana also revealed ∼30 kb outside ToxhAT that was ∼50% identical and partially overlapped the DNAs shared between P. tritici-repentis *and* P. nodorum (Fig. 2A [inclusive of genes PTRG-04892 to PTRG-04900]). This indicated that a region outside ToxhAT was potentially also horizontally transferred between B. sorokiniana and P. tritici-repentis. However, a pairwise alignment of this region showed no evidence of extensive RIP-like mutations that could account for the pairwise nucleotide differences observed between these two species (Fig. S4). Furthermore, we could identify the same region in *toxa^−^* isolate B. sorokiniana ND90Pr as well as in other closely related *Bipolaris* spp. (Fig. S5). We conclude that this region was not part of a horizontal transfer of ToxhAT into B. sorokiniana and instead represents a region of synteny between the two species.

10.1128/mBio.01515-19.4 FIG S4 Partial segment of the 30 kb region of shared sequence similarity between P. tritici-repentis and B. sorokiniana. This region is immediately upstream of ToxhAT in P. tritici-repentis. The alignment is wrapped ∼1,000 bp to aid visualization. The green bars in the “Identity” plot show alignment positions where the nucleotides match. The names of the isolates in the alignment are shown on the far left; the first sequence represents P. tritici-repentis 1C-BFP, and the second represents B. sorokiniana CS10. Annotated genes are shown in red or pink bars below the sequence. Colored vertical lines on the aligned sequences show differences between the two sequences. Unlike the alignments shown in Fig. S3, there are no recognizable G (yellow) → T (green) or C (purple) → A (red) transitions, which indicates that RIP is not responsible for the observed differences between these two sequences. The pairwise nucleotide identity of the ∼7 kb of aligned sequence shown here is 59.7%. Download FIG S4, PDF file, 0.4 MB. Copyright © 2019 McDonald et al. 2019 McDonald et al. This content is distributed under the terms of the Creative Commons Attribution 4.0 International license.

10.1128/mBio.01515-19.5 FIG S5 Closeup view of the ∼30 kb of DNA similarity shared between P. tritici-repentis and B. sorokiniana outside ToxhAT. This region directly flanks ToxhAT in P. tritici-repentis but is located ∼70 kb away from the 5′ ToxhAT TIR in B. sorokiniana (shown in Fig. 2A). Annotated genes were aligned and are indicated by solid green (*Bipolaris*) or blue (*Pyrenophora*) arrows. “%ID” indicates percent pairwise nucleotide identity between different isolates of the same or closely related species. The upper panel shows four *Bipolaris* genomes, including one each from B. sorokiniana CS10 and ND90Pr and one assembly each from B. maydis and B. oryzae. The lower panel shows four genomes of P. tritici-repentis. The red arrows drawn between B. sorokiniana and P. tritici-repentis show the pairwise percent nucleotide identity for the alignments between B. sorokiniana CS10 and P. tritici-repentis 1C-BFP. Dashed green boxes indicate positions of similarity between B. sorokiniana and P. tritici-repentis but with no annotated genes in B. sorokiniana. These data demonstrate that this region is syntenic between the two species as well as syntenic in other close relatives of B. sorokiniana (that do not carry ToxhAT). The data also show that the pairwise sequence identity is higher between the different *Bipolaris* spp. than it is between B. sorokiniana and P. tritici-repentis. The high degree of similarity between these two regions is therefore not related to the HGT of ToxhAT. Download FIG S5, PDF file, 0.03 MB. Copyright © 2019 McDonald et al. 2019 McDonald et al. This content is distributed under the terms of the Creative Commons Attribution 4.0 International license.

### Individual components of ToxhAT are found in other Pleosporales.

After careful manual annotation of ToxhAT, we conducted full *de novo* repeat prediction and annotation with the REPET pipeline (38, 39). The total proportion of each genome annotated as repeats is shown in Fig. S6. The nonredundant repeat library generated by REPET included the manually annotated ToxhAT transposon from B. sorokiniana CS10, named DTX-comp_CS10_RS_00, and a second nearly full-length version from P. nodorum, named DTX-incomp-chim_SN2000-L-B14-Map1. These two sequences were used to identify all instances of ToxhAT within each genome listed in Table 1 and in P. tritici-repentis 1C-BFP. A total of 195 instances of ToxhAT were annotated in these seven isolates, 183 (∼94%) of which we were able to successfully align to the CS10 ToxhAT sequence (Fig. S7). This alignment showed distinct areas within ToxhAT that were found in high abundance within these genomes, primarily overlapping *CS10_08.708*, *CS10_08.709*, and the Patatin-like gene (Fig. S7). A summary of the total number of identified ToxhAT instances is presented in Table 2. These data show that most of the annotations represent fragments, with the median length ranging from 176 to 295 bp, representing approximately 1% of the total length of ToxhAT.

**TABLE 2:** Summary of both partial and full-length ToxhATs identified by the repeat annotation pipeline in each isolate

Species and isolate	*n*^*b*^	Max length^*c*^	Min length^*c*^	Avg length^*d*^	Med length^*d*^
B. sorokiniana					
CS10	42	14,079	28	831	251
CS27^*a*^	22	1,552	26	437	179
WAI2406	35	14,082	28	935	225
WAI2411	41	14,072	28	891	250

P. tritici-repentis					
PTR1C-BPF	29	20,213	32	1,081	188

P. nodorum					
SN15	18	11,153	54	1,219	295
Sn79-1087^*a*^	8	537	99	266	261

a *toxa^−^* isolate.

b *n*, total number of ToxhAT annotations identified by the repeat annotation pipeline within the genome.

c Maximum (Max) or minimum (Min) length of ToxhAT in base pairs identified in the genome.

d Average (Avg) or median (Med) length of ToxhAT in base pairs identified in the genome.

10.1128/mBio.01515-19.6 FIG S6 Summary of transposon content of each genome represented as the percentage of total genome size. Transposons are classified according to the Wicker nomenclature into Orders (each column) and further classified into Superfamilies using color within each column. (A) Percent coverage of type I transposons found in each genome assembly. The B. sorokiniana isolates shown are CS10 (CS10_TA), CS27 (CS27_TA), WAI2406 (WAI2406_TA) and WAI2411 (WAI2411_TA). The P. tritici-repentis isolate shown is 1C-BFP (PTR_TA). The P. nodorum isolates shown are SN15 (SN15_TA), Sn2000 (SN2000_TA), Sn4 (SN4_TA), and Sn79-1087 (SN79_STA). (B) Percent coverage of the genome for type II transposons found in the same isolates. Download FIG S6, PDF file, 0.02 MB. Copyright © 2019 McDonald et al. 2019 McDonald et al. This content is distributed under the terms of the Creative Commons Attribution 4.0 International license.

10.1128/mBio.01515-19.7 FIG S7 Aligned ToxhAT instances from all genome sequences used in this study. REPET identified 205 instances of ToxhAT in total, and 183 could be mapped to the CS10 ToxhAT using the Geneious inbuilt mapper. ToxhAT from CS10, which was used as the reference for mapping the sequences below, is shown at the top of the alignment in yellow. The annotated genes from ToxhAT are shown in the yellow annotation rectangles. The blue “coverage” bar at the top of the alignment shows where the majority of sequences mapped. Each sequence at the bottom of the alignment was extracted from the isolate, and the chromosome is indicated in the name. Download FIG S7, PDF file, 1.3 MB. Copyright © 2019 McDonald et al. 2019 McDonald et al. This content is distributed under the terms of the Creative Commons Attribution 4.0 International license.

The large number of partial ToxhAT annotations in *toxa^−^* isolates suggested that some regions may be repetitive elements, independent of ToxhAT. To investigate this further, we performed tBLASTn queries using the NCBInr database and the Dothideomycetes genomes available at JGI MycoCosm. In both searches, the hAT transposase, MFS transporter, and Patatin-TPR genes had over 500 partial hits with an E value of less than 1e−10 (Fig. S8A and B). Within the JGI Dothideomycetes database search, *CS10_08.708* had 139 hits, *CS10_08.709* had 85 hits, and the *Tc1* transposase had 263 hits (E value, <1e−10). The small gene *CS10_08.714* had the lowest number of hits, with only four in total across both databases (excluding known instances of ToxhAT). Hits for the *ToxA* gene itself represented mostly distant (<50% identical) homologs, previously described as *ToxA*-like or ToxA* in various *Bipolaris* spp. (40). A short summary of the top hits from both database searches is presented in Table 3.

**TABLE 3:** Summary of top BLAST hits excluding known examples of ToxhAT in *P. tritici-repentis*, *P. nodorum*, and *B. sorokiniana*^*a*^

Query	Sequence length (aa)	Database	Genome/ BLAST description	Class^*c*^/ order	Location or accession no.	% identity	% query coverage	E value	Bit score
CS10_08.708	84	JGI	Didymella exigua CBS 183.55 v1.0^*b*^	Pleosporales	Didex1|scaffold_87:8242–8451	85.7	83.3	9.89E−36	305
			Didymella exigua ArDII	Pleosporales	Ascra1|scaffold_164:46459–46707	71.1	98.8	9.28E−33	284
			Lizonia empirigonia CBS 542.76 v1.0	Dothideomycetes^*c*^	Lizem1|scaffold_85:18354–18605	77.1	72.6	2.60E−34	251

		NCBI	Alternaria alternata DNA, AMT genes region, strain: NBRC 8984^*b*^	Pleosporales	AB525198.1	90.5	100	3.23E−34	129
			Bipolaris maydis clone FNFP145-M02, complete sequence	Pleosporales	AC277024.1	67.3	65.5	4.28E−13	69.7
			Bipolaris maydis clone FNFP209-G23, complete sequence	Pleosporales	AC277374.1	67.3	65.5	4.28E−13	69.7


CS10_08.709	55	JGI	Didymella exigua CBS 183.55 v1.0^*b*^	Pleosporales	Didex1|scaffold_87:7965–8131	90.7	78.2	2.63E−27	201
			Phoma multirostrata 7a v1.0	Pleosporales	Phomu1|scaffold_34:319170–385826	81.4	78.2	7.06E−24	183
			Lizonia empirigonia CBS 542.76 v1.0	Dothideomycetes^*c*^	Lizem1|scaffold_39:422775–422939	69.1	100	1.10E−18	177

		NCBI	Alternaria alternata DNA, AMT genes region, strain: NBRC 8984^*b*^	Pleosporales	AB525198.1	95.3	78.2	3.48E−18	82.4
			Bipolaris maydis ATCC 48331 hypothetical protein mRNA	Pleosporales	XM_014219256.1	69.6	100	3.85E−14	69.7
			Bipolaris maydis clone FNFP145-M02, complete sequence	Pleosporales	AC277024.1	69.6	100	1.04E−13	69.7


CS10_hAT	647	JGI	Decorospora gaudefroyi v1.0	Pleosporales	Decga1|scaffold_269:17873–19837	90.6	56.1	0.00E+00	1653
			Ophiobolus disseminans CBS 113818 v1.0	Pleosporales	Ophdi1|scaffold_7:902547–1437652	87.9	56.1	0.00E+00	1614
			Pyrenophora tritici-repentis 1C-BFP	Pleosporales	Pyrtr1|supercontig_1.22:2970–154460	83.2	56.1	0.00E+00	1496

		NCBI	Alternaria alternata DNA, AMT genes region, strain: NBRC 8984	Pleosporales	AB525198.1	53.5	88.3	0.00E+00	612
			Alternaria alternata TLS-S1-3 transposase pseudogene sequence	Pleosporales	AB236735.1	44.4	88.1	6.14E−164	507
			Alternaria alternata TLS-S1-2 transposase pseudogene sequence	Pleosporales	AB236734.1	44.4	88.1	6.14E−164	507


CS10_ToxA	178	JGI	Cochliobolus heterostrophus C5 v2.0	Pleosporales	CocheC5_3|scaffold_2:2350132–2350380	54.6	24.7	1.44E−16	126
			Cochliobolus heterostrophus C4 v1.0	Pleosporales	CocheC4_1|scaffold_114:2535–2783	54.6	24.7	1.50E−16	126
			Cochliobolus heterostrophus C5 v2.0	Pleosporales	CocheC5_3|scaffold_2:2350132–2350380	36.8	21.3	1.44E−16	85

		NCBI	Bipolaris maydis strain C4 ToxA-like protein (TOXA) mRNA, complete	Pleosporales	KJ664925.1	43.1	78.7	4.92E−26	105
			Bipolaris maydis ATCC 48331 hypothetical protein mRNA	Pleosporales	XM_014216967.1	43.1	78.7	5.78E−26	105
			Bipolaris maydis strain C5 ToxA-like protein (TOXA) mRNA, complete	Pleosporales	KJ664924.1	43.1	78.7	9.98E−26	105


MFS	202	JGI	Setosphaeria turcica NY001 v2.0	Pleosporales	Settur3|scaffold_53:4328–83573	70.3	55.0	5.35E−51	419
			Setosphaeria turcica Et28A v2.0	Pleosporales	Settu3|scaffold_6:884922–885254	70.3	55.0	1.48E−50	419
			Trematosphaeria pertusa CBS 122368 v1.0	Pleosporales	Trepe1|scaffold_8:1918893–1919171	71.0	46.0	6.11E−44	372

		NCBI	Setosphaeria turcica Et28A hypothetical protein partial mRNA	Pleosporales	XM_008031524.1	68.1	57.4	1.35E−46	168
			Parastagonospora nodorum SN15 hypothetical protein (SNOG_12844),	Pleosporales	XM_001803010.1	64.9	46.5	1.22E−33	132
			Parastagonospora nodorum isolate Sn2000 chromosome 18 sequence	Pleosporales	CP022843.1	64.9	46.5	2.13E−33	133


CS10_08.714	85	JGI	Alternaria alternata SRC1lrK2f v1.0	Pleosporales	Altal1|scaffold_52:12656–12787	77.3	51.8	1.70E−15	170
			Ophiobolus disseminans CBS 113818 v1.0	Pleosporales	Ophdi1|scaffold_44:27494–27639	70.3	43.5	1.46E−11	128
			Ophiobolus disseminans CBS 113818 v1.0	Pleosporales	Ophdi1|scaffold_44:27494–27639	81.8	12.9	1.46E−11	48

		NCBI	Alternaria alternata hypothetical protein partial mRNA	Pleosporales	XM_018535381.1	77.3	51.8	2.41E−14	67.4
			Alternaria alternata FabD/lysophospholipase-like protein mRNA	Pleosporales	XM_018526331.1	77.3	51.8	3.66E−12	67


Patatin	936	JGI	Setomelanomma holmii CBS 110217 v1.0	Pleosporales	Setho1|scaffold_339:2–8483	79.6	17.3	0.00E+00	685
			Cochliobolus victoriae FI3 v1.0	Pleosporales	Cocvi1|scaffold_110:673–4261	77.1	18.2	0.00E+00	680
			Clohesyomyces aquaticus v1.0	Pleosporales	Cloaq1|scaffold_160:91821–95443	77.8	17.3	0.00E+00	677

		NCBI	Alternaria alternata FabD/lysophospholipase-like protein mRNA	Pleosporales	XM_018526331.1	73.2	100	0.00E+00	1383
			Bipolaris victoriae FI3 hypothetical protein partial mRNA	Pleosporales	XM_014696900.1	73.2	92.3	0.00E+00	1348
			Parastagonospora nodorum SN15 hypothetical protein (SNOG_12454)	Pleosporales	XM_001802625.1	70.3	93.9	0.00E+00	1258


Tc1	75	JGI	Phoma tracheiphila IPT5 v1.0	Pleosporales	Photr1|scaffold_21:57505–57729	62.7	100	3.31E−24	228
			Phoma tracheiphila IPT5 v1.0	Pleosporales	Photr1|scaffold_93:22667–22891	61.3	100	5.80E−23	219
			Setomelanomma holmii CBS 110217 v1.0	Pleosporales	Setho1|scaffold_126:700–924	52.0	100	3.45E−19	192

		NCBI	Rasamsonia emersonii CBS 393.64 hypothetical protein partial	Eurotiomycetes^*c*^	XM_013470328.1	40.0	100	4.95E−11	60.5
			Rasamsonia emersonii CBS 393.64 hypothetical protein partial	Eurotiomycetes^*c*^	XM_013469195.1	40.0	100	5.27E−11	60.5
			Rasamsonia emersonii CBS 393.64 hypothetical protein partial	Eurotiomycetes^*c*^	XM_013473453.1	44.9	92.0	5.61E−11	59.3

a Only the top 3 BLAST hits are shown sorted according to the bit score, which is a measure of pairwise sequence similarity. aa, amino acids.

b Data indicate hits of interest with >85% pairwise nucleotide identity and >78% query coverage.

c Class data are indicated for species outside the Dothideomycetes or for which the order is unknown.

10.1128/mBio.01515-19.8 FIG S8 (A) Visual summary of the BLAST results from NCBI nr database. Each point represents a single BLAST hit, and the colors indicate different species. Hits to known examples in P. nodorum, P. tritici-repentis, and B. sorokiniana were excluded from this figure. (B) The data are presented as described for panel A, but the results are from the JGI Dothideomycetes database. Download FIG S8, PDF file, 2.2 MB. Copyright © 2019 McDonald et al. 2019 McDonald et al. This content is distributed under the terms of the Creative Commons Attribution 4.0 International license.

The hits with the highest identity were in a single Alternaria alternata strain, NBRC 8984 (GenBank accession no. AB525198.1), for two contiguous open reading frames, *CS10_08.708* and *CS10_08.709*. The pairwise identity for these two genes exceeded 90%, and the genes are colocalized in A. alternata NBRC 8984. This strain was also the highest-identity hit in NCBI for the hAT-transposase. A. alternata is a well-known plant pathogen that has a broad host range and is also a member of the Pleosporales. In the JGI database, a fungal isolate collected from leaf litter, Didymella exigua CBS 183.55 v1.0, also produced colocalized hits for *CS10_08.708* and *CS10_08.709* with identity greater than 85%, indicating that these two predicted genes may represent a single repetitive unit. This species is also classified in the Pleosporales (41). The third species identified with >90% sequence identity for the hAT transposase was Decospora gaudefroyi, again classified in the Pleosporales as a salt-tolerant marine fungus (42). Overall, the large number and breadth of hits across different fungal species confirmed our hypothesis that the individual coding regions of ToxhAT are part of repetitive elements broadly found within the Pleosporales. This indicates a common evolutionary origin of these repetitive coding regions within this fungal Order.

### The presence/absence polymorphism of *ToxA* is much larger than the extent of HGT.

**FIG 3:**
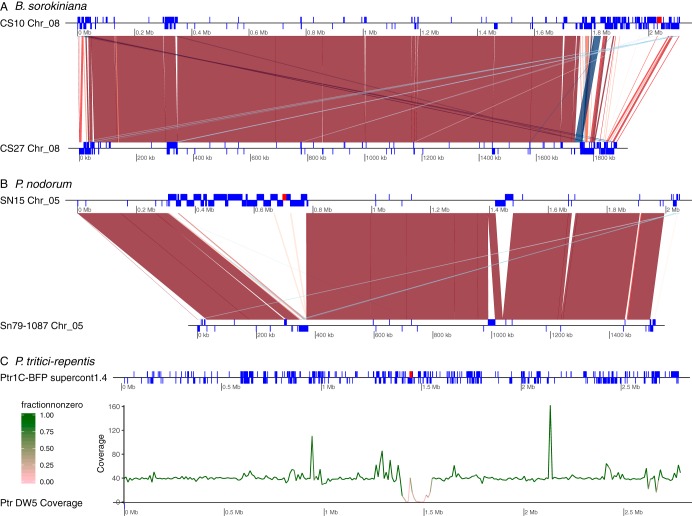
Genomic context of the ToxhAT-containing region (red box) in each of the three species in comparison to *toxa^−^* isolates. (A) Lastz alignment of the homologous chromosome between B. sorokiniana *ToxA^+^* isolate CS10 and *toxa^−^* isolate CS27. Blue blocks drawn on the chromosome maps (black lines) represent the location of annotated transposons within each genome. Red ribbons drawn between the two isolates represent syntenic alignments found in Lastz that showed more than 70% identity and were greater than 2 kb in length. Blue ribbons drawn between the two isolates show inversions between the two genomes. (B) Lastz alignment of the homologous chromosome between P. nodorum *ToxA^+^* isolate SN15 and *toxa^−^* isolate Sn79-1087. The coloring scheme is the same as that used in panel A. (C) Isolate *Ptr*1C-BFP with repeat regions shown in the blue blocks along the chromosome (black line). The average Illumina coverage for 10 kb windows across the chromosome is indicated at the bottom. The color of the line corresponds to the proportion of bases within the 10 kb window that had nonzero coverage.

We showed above that the extent of shared DNA differ between different pairwise comparisons of the three species harboring ToxhAT. This does not address the unknown size of the presence/absence polymorphism maintained in these species. To investigate this, the homologous *toxa^−^* and *ToxA^+^* chromosomes were aligned (Fig. 3). For P. tritici-repentis, where no long-read data for a *toxa^−^* isolate were available, short reads from isolate DW5 were aligned against the assembly of P. tritici-repentis 1C-BFP. The large spike in coverage for DW5 within the deleted region corresponds to a 7 kb TIR transposon, most likely from the Tc1-Mariner superfamily, which is found near ToxhAT (DTX-incomp-chim_Ptr-L-B62-Map1_reversed). Both of the chromosomes containing *ToxA* in isolates P. nodorum SN15 and B. sorokiniana CS10 contain telomeric repeats and are complete chromosomes. This shows that the absence polymorphism spans several thousand kilobases in all three species (Fig. 3). Using the last known homologous regions from the whole-chromosome alignments, the absence alleles in B. sorokiniana CS27, P. nodorum Sn79-1087, and P. tritici-repentis DW5 were estimated to span ∼239 kb, ∼467 kb, and ∼150 kb, respectively.

To compare the size of the presence/absence polymorphism of *ToxA* with the sizes of two other well characterized necrotrophic effectors, we examined the location of *SnTox3* and *SnTox1* in P. nodorum SN15 (43). These two effectors also exist as a presence/absence polymorphism in this species but have no known history of HGT. Sn*Tox3* is the last annotated gene on chromosome 11 in isolate SN15, and the absence polymorphism approximately corresponds to the 7 kb tail of this chromosome. This absence encompasses annotated SN15 genes SNOG_08981 (*Tox3*) to SNOG-08984 (Fig. S9A). The end of chromosome 11 in the assembly of Sn79-1087 (which lacks *SnTox3*) contains telomeric repeats (data not shown), which suggests that the absence of the 7 kb is not due to an incompletely assembled chromosome. The absence allele of *SnTox1* is even smaller, spanning ∼3 kb on chromosome 6 of *SN15.* At this locus, there is a unique insertion of ∼1.3 kb which is present only in Sn79-1087 (Fig. S9B). These data demonstrate that the absence allele of the horizontally transferred *ToxA* gene is much larger than absence alleles of other known effectors and highlight potential genome instability after HGT events.

10.1128/mBio.01515-19.9 FIG S9 (A) Alignment of the presence/absence polymorphism of *SnTox3* in P. nodorum on chromosome 11. The end of the chromosome in each isolate is indicated by the black arrows. The *SnTox3* effector gene is the last gene on the chromosome (SNOG_08981) in isolate SN15. That gene and the three neighboring genes are all missing from *toxa*^−^ isolate Sn79-1087. Red blocks indicate where the two homologous chromosomes begin to align with each other. Both assembles contain telomeres on this chromosome end, indicating that the absence of the last four genes is not due to an incomplete chromosome. (B) Alignment of the presence/absence of polymorphism of *SnTox1* in P. nodorum in chromosome 6. Gray color in the alignment indicates where the two sequences are identical. Black color indicates differences between the two alignments. Percent nucleotide identity is shown at the top of the alignment. *SnTox1* (SNOG_20078) is absent from *toxa^−^* isolate Sn79-1087. This deletion spans ∼3,000 bp in isolate SN15, but there is a unique insertion of 1.3 kb in isolate Sn79-1087. Unlike *SnTox3* (shown at the top), this deletion event is not near a telomere in either isolate. Download FIG S9, PDF file, 0.5 MB. Copyright © 2019 McDonald et al. 2019 McDonald et al. This content is distributed under the terms of the Creative Commons Attribution 4.0 International license.

### Evidence of mobility of ToxhAT in B. sorokiniana.

**FIG 4:**
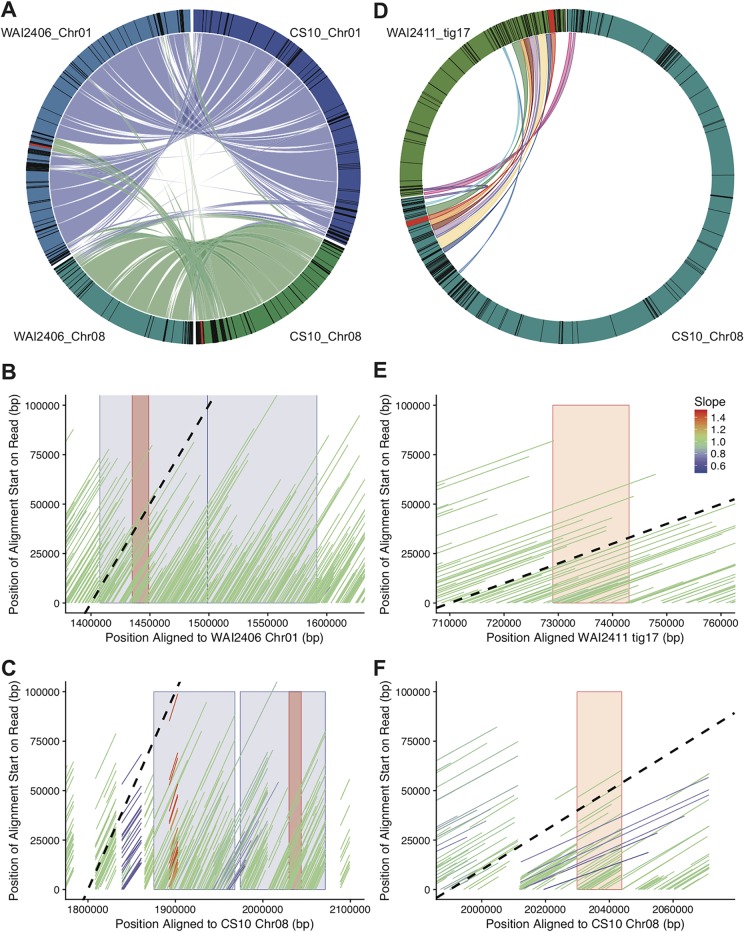
ToxhAT within B. sorokiniana is mobile in two distinct ways. (A) Alignment of chromosomes 01 and 08 of CS10 against WAI2406 homologous chromosomes. The ToxhAT (red line/box drawn on outer circle) is located on Chr01 in WAI2406 along with a large region of repeat-rich DNA (black boxes in outer circle). (B) The corrected and trimmed WAI2406 Nanopore reads aligned to the *de novo* version of itself. The black dotted line shows a slope value of 1, which indicates that the read aligned base per base to the chromosome shown on the *x* axis. Reads with a slope value different from 1 are reads that have been mapped discontinuously (i.e., with large insertions or deletions). The blue blocks show the translocated DNA, found in chromosome 08 in CS10 but in chromosome 01 in WAI2406. The red box indicates the position of ToxhAT. (C) The data correspond to the same reads as those described for panel B but represent alignment to chromosome 08 of CS10. Note that the read alignment is not continuous and breaks at the translocation edges. (D) Alignment of chromosome 08 from CS10 against tig17 from WAI2411. (E) The data are presented as described for panel B but represent isolate WAI2411. The red block shows the position of ToxhAT. (F) The data are presented as described for panel C but represent isolate WAI2411. Note that no reads with a slope value of 1 extend beyond the ToxhAT itself (red box).

The finding of intact TIRs in B. sorokiniana CS10 suggested that ToxhAT in this species may remain mobile. To investigate this, we resequenced two additional *ToxA^+^* isolates of B. sorokiniana (WAI2406 and WAI2411). ToxhAT was found in different genomic locations in the two genomes compared to isolate CS10, where ToxhAT is located near the end of chromosome 08 (Fig. 4A). For WAI2406, ToxhAT and the surrounding ∼200 kb of repeat-rich DNA were found imbedded in the middle of chromosome 01 (Fig. 4A to C). This has led to an increase in the size of WAI2406’s chromosome 01, which is ∼4.0 Mbp compared to CS10’s PacBio assembled chromosome 01 size of ∼3.8 Mbp. To confirm that this translocation was not a misassembly, we aligned the corrected Nanopore reads from Canu to both the CS10 and WAI2406 assemblies (Fig. 4B and C). These reads aligned, with a slope of 1, to chromosome 01 in isolate WAI2406, with single reads clearly spanning the breakpoints on both sides of the translocation. These same reads from isolate WAI2406 did not align well to chromosome 08 in isolate CS10.

For isolate WAI2411, ToxhAT assembled to a small contig (∼776 kb) that has homology to chromosome 02 and chromosome 08 of the PacBio assembled CS10 (data not shown). While it remains unclear if this small scaffold is a part of chromosome 02 or chromosome 08, the flanking DNA on either side of ToxhAT was conserved but shuffled in order (Fig. 4D). ToxhAT was found to be inverted and in a different position from that in chromosome 08 in CS10. The breakpoints of the inversion were precisely from TIR to TIR of ToxhAT. Again, we aligned the corrected nanopore reads to isolate WAI2411 (self) and isolate CS10. The self-alignment showed reads that clearly crossed the breakpoints of the inversion/transposition (Fig. 4E); however, these same reads did not map contiguously to the CS10 assembly (Fig. 4F). As a secondary confirmation, we used BLASTn to identify all reads that contained *ToxA* and generated a multiple-sequence alignment (Fig. S10). Twenty-nine single-molecule reads were aligned that spanned ToxhAT and continued into both flanking regions. These flanking regions aligned well to WAI2411’s contig 17, confirming the inversion of ToxhAT precisely at the TIRs (Fig. 4E). We postulated that this inversion could represent an active transposition event, in which case there might be signature target-site duplications (TSDs) made by the transposase. However, neither this inversion nor any other instance of ToxhAT in any sequenced isolate was flanked by identifiable target-site duplications.

10.1128/mBio.01515-19.10 FIG S10 Pairwise nucleotide alignment of raw MinION reads for B. sorokiniana isolate WAI2411 that contain the ToxA gene sequence. Alignment is wrapped approximately every 30 kb to aid visualization. The average pairwise nucleotide identity for this alignment is 96.4%. Download FIG S10, PDF file, 1.5 MB. Copyright © 2019 McDonald et al. 2019 McDonald et al. This content is distributed under the terms of the Creative Commons Attribution 4.0 International license.

## DISCUSSION

As shown in previous studies, the *ToxA* gene and surrounding noncoding DNA are highly conserved between these three species (17, 20, 30). We extend this knowledge by defining flanking TIRs that give this region the structural features of a type II DNA transposon, defined here as ToxhAT. These TIRs and the enclosed 14.3 kb are conserved in all three fungal species, indicating that ToxhAT had a common evolutionary origin prior to HGT. The finding of homologous DNA shared between P. nodorum and P. tritici-repentis outside these TIRs indicates that the HGT event between these two species did not involve ToxhAT alone but included ∼63 kb of flanking DNA. In contrast, homology between these two species and B. sorokiniana breaks precisely at the ToxhAT TIRs. Within B. sorokiniana, there is strong evidence that ToxhAT and the surrounding repeat-rich DNA remain mobile within the genome. This finding contrasts with two previously published long-read assemblies of P. nodorum and P. tritici-repentis, which show *ToxA* and the surrounding horizontally transferred (HT) DNA on the same chromosome (23, 29, 30). The individual coding regions found within ToxhAT appear to be part of repetitive elements in other Dothideomycetes. However, the breadth of hits gives no indication of whether any single species could have assembled ToxhAT as a unit before horizontal transfer between these three wheat pathogens.

### ToxhAT is structured like a type II DNA transposon, but mobility may be facilitated by recombination.

By identifying conserved TIRs in all three pathogens, we describe a unit of DNA that has the structural features of a type II DNA TIR transposon. In B. sorokiniana isolate WAI2411, ToxhAT itself is inverted precisely at the TIRs. This inversion bounded precisely by these structural features suggested that this putative transposon may remain active. However, extensive searches of the flanking DNA in all three species did not reveal TSDs typical of other TIR transposases (44, 45). TSDs are created by a transposase when it cuts at its target site, usually a short sequence ranging in length from 2 to 11 bases (34). Most transposases make an uneven cut which, after DNA repair, leads to a duplication of the target site on either side of the inserted transposon (34). The absence of TSDs in WAI2411 suggests that the inversion seen in isolate WAI2411 was not facilitated by an active transposition event. An alternative mechanism that could explain the inversion is intrachromosomal recombination between these structural features (25). In the absence of TSDs, we consider this mechanism a strong alternative to explain the movement of ToxhAT in WAI2411.

While WAI2411 showed a relatively small genomic rearrangement, the other resequenced B. sorokiniana isolate, WAI2406, contained a large segmental movement of 200 kb from one chromosome to another. Similar interchromosomal translocations were observed previously in the fungal plant pathogens Verticillium dahliae, Magnaporthe oryzae, and Colletotrichum higginsianum (46–48). While all three studies demonstrated that translocations occur in regions where transposons are prevalent, only the study by Faino et al. (47) in the asexual species V. dahliae was able to show homologous transposons/DNA sequence at the translocation breakpoints. These breakpoints in V. dahliae implicate homologous recombination as the mechanism underpinning genome plasticity in this species (47). In M. oryzae and C. higginsianum, transposons are associated with translocation events, but it remains unclear if these translocations occurred over regions with high sequence identity (46, 48). Similarly, in B. sorokiniana, whole-chromosome alignment of chromosome 08 and chromosome 01 in isolate WAI2406 did not show high sequence identity in the regions outside the translocation breakpoints on either chromosome. However, the regions near these breakpoints are enriched in transposon annotations. A key difference between the three fungal species examined in this study and *Verticillium* is their sexual life cycle. Meiotic recombination could obscure the precise translocation boundaries and is also a prerequisite for RIP. We postulate that in sexual fungal species, AT-rich regions, with otherwise limited sequence identity, may undergo “homologous” interchromosomal recombination events like those observed in B. sorokiniana. Supporting this hypothesis is a recent study on Epichloë festucue that leveraged Hi-C data to build a contact map of DNA within the nucleus (49). The Hi-C sequencing technique shows the frequency at which different regions of a genome interact with each other in the nucleus (50). For example, DNA fragments that are physically close to each other, on the same chromosome, have a higher frequency of interaction than sequences on different chromosomes (49). A study by Winter et al. showed that AT-rich regions, usually in the form of heavily RIPed transposon islands, on different chromosomes had significantly higher interactions with each other than non-AT-rich regions (49). These data suggest that in sexual fungi, where RIP is active, AT-rich islands are associated with each other in three-dimensional (3D) space. We postulate that AT-rich regions in B. sorokiniana, like those in E. festucue, also associate with each other within the nucleus, which provides the physical proximity required for interchromosomal recombination events.

The opportunity to examine interchromosomal transfer of ToxhAT extends beyond B. sorokiniana. Chromosomal movement of the *ToxA* gene was also observed in P. tritici-repentis (51). In that study, pulsed-field gel electrophoresis followed by Southern hybridization was used to show that *ToxA* was found on chromosomes of different sizes in P. tritici-repentis. Going further, the authors showed that, in at least one isolate, *ToxA* was on a chromosome different from the *ToxA* location in reference isolate 1C-BFP (51). While that study probed for the *ToxA* gene alone, we consider it likely that ToxhAT or a larger chromosomal segment was translocated, similarly to what was observed in B. sorokiniana. Further long-read assembly coupled with Hi-C data from both P. tritici-repentis and B. sorokiniana, ideally from a sexual cross of two previously sequenced isolates, is required to systematically reconstruct the level of sequence identity or other genome features that facilitate interchromosomal translocations.

### ToxhAT resides in an accessory genomic region in all three species.

The analysis of the syntenic relationship between *ToxA^+^* and *toxa^−^* chromosomes within each species showed that the absence of ToxhAT is coincident with the absence of large (>100 kb) chromosomal segments. The DNA composition of these regions fits well within the definition of the “lineage-specific” or “accessory” regions described in pathogenic fungi, where virulence genes are found nested within transposon-rich regions of the genome (52–55). This genome structure is hypothesized to facilitate the rapid adaptation of fungal pathogenicity genes, often referred to as the “two-speed” or “two-compartment” genome (56, 57). These data further show that the absence polymorphism does not coincide with the exact boundaries of HGT. This is most clearly seen in P. nodorum, where fragments of the horizontally transferred DNA are scattered across a 300 kb region. However, the entire region, extending well beyond the horizontally transferred fragments, is missing from *toxa^−^* isolate Sn79-1087. This leads to an interesting issue concerning whether the horizontal acquisition of ToxhAT precipitated the expansion of transposons within this region. Our data for *SnTox3* and *SnTox1* in P. nodorum suggest that this may be the case, whereby the absence alleles for these well-characterized effectors span only a few kilobases. Unfortunately, similar comparisons were not possible in B. sorokiniana due to a lack of known effectors or in P. tritici-repentis, where the only other known effector, *ToxB*, is present in multiple copies in the genome (58). Intrachromosomal recombination is also a possible mechanism to generate these large absence polymorphisms. In many model organisms, such as *Drosophila*, yeast, and human cell lines, large segmental deletions were previously found to be facilitated by ectopic recombination between tandemly arrayed repeat sequences (59, 60). This mechanism is particularly interesting in the context of HGT, as these recombination events often result in the formation of circular extrachromosomal DNA (61, 62).

### The origins of ToxhAT remain obscure.

Since the discovery of *ToxA* in the genome of P. nodorum, the evolutionary origin of this gene has been a topic of debate (20, 23, 63, 64). To date, P. nodorum remains the species with the highest known *ToxA* sequence diversity. This diversity underpins the prevailing hypothesis that *ToxA* has had the most time to accumulate mutations and therefore has resided in the genome of this organism longest (20, 63). The discovery of *ToxA* in B. sorokiniana and the characterization of the conserved 74 bp TIRs in all three species strongly suggest that ToxhAT had a single evolutionary origin in all three species. The ToxhAT TIRs in B. sorokiniana define the exact boundaries of the HGT event, where sequence identity with the other two species abruptly ends. In contrast, the HGT between P. tritici-repentis and P. nodorum included ToxhAT and 63 kb of flanking DNA. In P. tritici-repentis 1C-BFP, this flanking DNA remains contiguous; in P. nodorum, however, this same DNA is fragmented and partially duplicated across a 370 kb island of RIPed transposable elements (TEs). Together, these data suggest that ToxhAT was horizontally transferred in two separate events both with and without flanking DNA.

We propose two opposing models to explain the HGT of ToxhAT between the three species. In the first model, we assume a population genetic perspective where the longer the DNA has resided in the genome, the more fragmented and dispersed the HT DNA becomes. In this model, P. nodorum would be the donor of ToxhAT along with the flanking DNA to P. tritici-repentis. This is based on our observation that the flanking DNA outside ToxhAT is highly fragmented and duplicated in P. nodorum, indicating this species has had more time to accumulate these changes. This model assumes that the P. nodorum donor isolate once had a contiguous stretch of DNA as observed in P. tritici-repentis. In this model, we also postulate that ToxhAT was most recently acquired by B. sorokiniana due to its relatively compact form and conservation of structural features. Again, for this model to hold, we must assume that an intact form of ToxhAT exists in the population of P. nodorum or P. tritici-repentis that could act as a donor to B. sorokiniana. Our second model assumes that changes can accumulate more rapidly in transposon-rich regions and therefore sequence diversity in these regions is not a good indication of evolutionary time. In this model, the intact version of ToxhAT observed in B. sorokiniana would represent an ancestral version. This is the minimal unit of HT DNA, bounded by conserved TIRs. In this model, we propose that the first HGT event was from B. sorokiniana to P. tritici-repentis, based on the identical *ToxA* sequences that they share. Then, in a second HGT event, P. tritici-repentis would have been the donor of the large segment of DNA inclusive of ToxhAT to P. nodorum. The HT DNA flanking ToxhAT was then subject to rapid decay and duplication in P. nodorum, due to its proximity to transposable elements. This model does not provide a good explanation of why the rapid decay is not also observed in the other two species.

In order to differentiate between these two models, we suggest long-read sequencing across the broadest possible sample times and locations for both *ToxA^+^* and *toxa^−^* isolates in all three species. The first goal of this sequencing would be to systematically characterize the sequence diversity of ToxhAT variants all three species. The presence of any “intact” ToxhAT sequences coupled with overall higher ToxhAT sequence diversity in isolates of P. nodorum and P. tritici-repentis would support the first model. Second, we suggest searching the sequence of *toxa*^−^ isolates for the HT DNA flanking ToxhAT in P. nodorum and P. tritici-repentis. For example, if this region were present in a *toxa^−^* P. nodorum isolate, this would identify P. nodorum as the donor of ToxhAT and its flanking DNA to P. tritici-repentis. Determining the direction of transfer between B. sorokiniana and the other two species is more challenging, given the overall high sequence conservation of ToxhAT. Support for the model where B. sorokiniana harbors the ancestral ToxhAT could come from sequence data that show that ToxhAT in this species has a level of sequence diversity similar to that seen with the other two species.

While we have presented two models above that describe the history of HGT between these species alone, results of the BLAST searches conducted on the coding regions annotated in B. sorokiniana indicate that there are other species which may harbor highly identical components of ToxhAT. One standout isolate is A. alternata strain NBRC8984, which carries two genes that are 90% and 95% identical to *CS10_08.708* and *CS10_08.709*, respectively. Similarly to their arrangement in ToxhAT, these two coding sequences also neighbor each other in A. alternata NBRC8984. These hits within ToxhAT are by far the most similar sequences found in a species that is not reported to carry ToxA. These high-identity hits also included some nonpathogenic and marine species also found within the Pleosporales. Collectively, the coding regions within ToxhAT had hundreds of hits across species representing several hundred million years of evolution. However, none of these coding regions have been characterized as repetitive or classified in a transposon family. Despite the ancient evolutionary history of transposons, the vast majority of described DNA transposons with TIRs are classified into only 10 superfamilies (34, 65). Our detailed characterization of ToxhAT highlights an opportunity to characterize novel transposon superfamilies in nonmodel fungi.

### Towards a mechanism; flanking noncoding DNA provides clues.

The tBLASTn results, coupled with a detailed structural characterization of ToxhAT, suggest that it is a mosaic of repetitive coding regions. We propose that ToxhAT was transferred horizontally as, or by, a transposon, with the fitness advantage of *ToxA* fixing these HGT events in three wheat-infecting species. Similarly, the horizontally transferred regions in the cheese-making *Penicillium* spp. were flanked by unusual *i* non-LTR retrotransposons (13). In the present study, it remained unclear whether ectopic recombination, rather than active transposition, is the mechanism that integrates the HT DNA into the recipient genome. Horizontal transfer of transposons (HTT) has been widely reported in eukaryotes since the early discovery of P elements in *Drosophila* (66, 67). The literature on this topic, however, seems to clearly divide HGT from HTT as two separate phenomena, the latter being much more common (68–70). Recent reports of the HTT between insects has used noncoding regions flanking horizontally transferred genes to demonstrate that a viral parasite, with a broad insect host range, is the vector for the horizontally transferred DNA (71). This report highlights how insights from noncoding regions can bring these studies closer to a mechanistic understanding of the HGT event (71, 72). Other studies which report HGT of secondary metabolite clusters into and between fungal species often rely on phylogenetic methods performed using coding regions alone to detect these events (73–76). While these studies focus on the biological significance of the coding regions, clues to a possible mechanism may remain in the surrounding noncoding DNA.

One limitation encountered with early Illumina-based genome assemblies was the inability to correctly assemble highly repetitive regions. Here we demonstrated with two long-read sequencing technologies that it is possible to assemble very large repetitive regions heavily affected by RIP. These assemblies allow us to look at the noncoding and transposon-rich regions that may be important for the movement or integration of HT DNA. Further population-scale long-read sequencing will enable further refinement of our understanding of the role that transposons play in facilitating adaptive gene transfer.

## MATERIALS AND METHODS

### Fungal culture and DNA extraction.

Fungal cultures were grown on V8-potato dextrose agar (PDA) media at 22°C under conditions of a 12-h light/dark cycle (77). Cultures ranging in age from 5 to 10 days were scraped from the surface of agar plates into water by the use of a sterile razor blade. These harvested cultures were freeze-dried for 48 h to remove all water. High-molecular-weight (HMW) DNA was extracted using a C-TAB phenol-chloroform method modified from Fulton et al. (1995) (78). Full details of our protocol, including gel images of the final DNA, are available at https://doi.org/10.17504/protocols.io.hadb2a6. DNA size was assessed by pulsed-field electrophoresis and DNA purity by examining 260/280 and 230/280 UV absorbance ratios on a NanoDrop spectrophotometer (Thermo Scientific, USA). The total quantity of DNA was measured using a Qubit fluorometer (Life Technologies, USA).

### Genome sequencing and assembly. (i) PacBio DNA sequencing.

Isolates SN15 (P. nodorum) and CS10 (B. sorokiniana; BRIP10943) were sequenced using Pacific Biosciences SMRT sequencing. Genomic P6 SMRT cell library preparations (15 to 20 kb) were made at the Ramaciotti Centre for Genomics (University of New South Wales [UNSW] Sydney, Sydney, Australia). Each library was sequenced on 7 SMRT cells by the use of P6-C4 chemistry on the PacBio RSII instrument (Pacific Biosciences, USA). Libraries were size selected on a Blue Pippen from 15-50 kb. Each isolate genome was assembled *de novo* using Canu v1.5 and a minimum read length of 2,000 bp (79). The Canu parameter “genomeSize” was set to 41 Mb for isolate SN15 and 35 Mb for isolate CS10. Canu assemblies were further corrected using SMRT Analysis package v2.3.0. First, the raw PacBio reads were mapped to the *de novo* assembly using blaser with the following settings: --seed = 1 --minAccuracy = 0.75 --minLength = 500 --forQuiver --algorithmOptions=‘-minMatch 12 -bestn 1 -minPctIdentity 65.0 –hitPolicy=randombest'. The resulting bam file was used as input for Quiver to call a new consensus sequence with the following settings: makeVcf=True, makeBed=True, enableMapQVFilter=T, minConfidence = 40, minCoverage-10, diploidMode=False (80).

**(ii) Nanopore DNA sequencing.** Resequencing of B. sorokiniana isolates CS27 (BRIP27), WAI2406, and WAI2411 and P. nodorum isolate Sn79-1087 was performed on Oxford Nanopore's MinION sequencer. R9.4 flow cells were used for sequencing, and a SQK-LSK08 1D library kit was used to prepare the libraries according to the manufacturer’s protocol. All DNA samples were purified using Agencourt AMPure beads prior to starting the 1D library preparation (Beckman Coulter, Inc., CA, USA). Genomes were assembled with Canu v1.5 or v1.6 with a minimum read length of 5 kb (79). *De novo* genome assemblies were corrected using the trimmed reads output from Canu. Trimmed reads were mapped to the genome with Minimap2 followed by correction with Racon (81). The output consensus sequence from Racon was used as the input for additional corrections steps performed iteratively up to five times. Sn79-1087 and CS27 assemblies were further refined using Pilon software; this correction was performed iteratively up to five times (82). Sn79-1087 Illumina data were taken from Syme et al. (21) and BRIP27 Illumina from McDonald et al. (17).

**RNA sequencing to aid annotation of long-read assemblies.** B. sorokiniana isolate CS10 was cultured for 10 days in a range of liquid growth media, including V8-juice broth (potato dextrose broth [PDB]) (77); PDB supplemented with the epigenetic modifier 5-azacytidine (150 μM) (83); minimal media (84); wheat extract-supplemented minimal media; and Fries 3 and Fries 3 modified media (85). Mycelia were harvested, and total RNA was extracted with a Zymo Research fungal/bacterial RNA miniprep kit. RNA quality assessment (Agilent Bioanalyzer), library preparation (strand-specific TruSeq v3), and Illumina RNA sequencing (MiSeq; 150-bp single-end reads) were performed at the Ramaciotti Centre for Genomics (UNSW Sydney, Sydney, Australia).

Long-read RNA-seq data were also generated using Oxford Nanopore's MinION sequencer. Total RNA extracted from mycelia cultured in Fries 3 medium was enriched for mRNA by the use of NEBNext oligo(dT) magnetic beads and concentrated by the use of Agencourt RNAClean XP magnetic beads (Beckman Coulter, Inc., CA, USA). RNA-seq and cDNA-PCR libraries were generated directly with SQK-RNA001 and SQK-PCS108 library preparation kits, respectively, and sequenced with R9.4 flow cells. Reads were base called with Albacore v2.0.2, quality filtered with Nanofilt, and subjected to error correction using the CS10 genome sequence with Proovread (default settings) (86). Error-corrected reads were filtered for reads corresponding to full-length transcripts using SQANTI (run with default settings) (87).

### Annotation of long-read assemblies.

Illumina RNA-seq data were used for gene prediction in both CS10 and CS27 B. sorokiniana isolates. Reads were trimmed with Trimmomatic v0.32 (parameters: -phred 33, ILLUMINACLIP TruSeq3- SE.fa:2:30:10, SLIDINGWINDOW:4:20, LEADING:20, TRAILING:20, and MINLEN:75) and aligned to the genomes using STAR v2.5 (parameters: --alignIntronMin 10, --alignIntronMax 1000, --twoPassMode Basic), and then transcript assembly was performed using StringTie v1.3.3 (default parameters, except for --f 0.3). StringTie transcripts were filtered for high-quality ORFs by the use of TransDecoder v5.0.2 (88–90).

Transcripts and aligned Illumina RNA-seq reads from all culture conditions were pooled for gene prediction. Pooled transcripts were used for gene prediction with CodingQuarry v2.0 (self-training Pathogen mode; default parameters) (91). Aligned reads and protein sequences from P. nodorum were used as evidence for gene prediction by BRAKER v2.0 (nondefault parameters --fungus, --prg gth) and GeMoMa v4.33 (default parameters) (92, 93). Gene predictions were combined with a nonredundant (nr) set of reviewed fungal Uniprot protein sequences and high-quality StringTie transcripts using EVidenceModeler (EVM), which generated a weighted consensus of all predicted gene models (Haas et al., 2008) (94). Evidence sources were weighted accordingly as follows: CodingQuarry > BRAKER ≥ GeMoMa > StringTie transcripts > nr fungal proteins. An assembly of MinION RNA-seq reads concordant with EVM gene models and StringTie transcripts was generated using PASA; this was used to update the EVM models (e.g., correcting intron-exon boundaries) and to annotate untranscribed regions (UTRs) (94). The completeness of these gene models was assessed using the BUSCO ascomycete database (32, 33). Complete gene annotations for B. sorokiniana isolates CS10 and CS27 are available in File S2 at https://github.com/megancamilla/Transposon-Mediated-transfer-of-ToxA/tree/master/S2_Bipolaris_Gff3.

### Annotation of transposons.

Transposons were identified *de novo* using the TEdenovo pipeline distributed as part of the REPET package v2.5 (38, 39). Long-read assemblies from the following species and isolates were used for *de novo* discovery: P. nodorum isolates Sn2000 and Sn4, assembled by Richards et al. (29), and SN15 (this study); P. tritici-repentis 1-C-BFP; and B. sorokiniana isolate CS10. Repbase v20.05 was used as the reference transposon database. TEs from each genome were combined into a common database according to the parameters set in Tedenovo_six_dnLibTEs_90_92.cfg. After combining the TEs, we manually added the coordinates of ToxhAT and named this TE “DTX-comp_CS10_RS_00.” Finally, TEs were annotated in each genome listed in Table 1 with the common TE database using the TEannot pipeline and settings available in the TEannot.cfg file available at https://github.com/megancamilla/Transposon-Mediated-transfer-of-ToxA. Transposons were automatically classified into three-letter codes based on the Wicker nomenclature (34, 95). (REPET pipeline configuration and run scripts, plus the repeat annotation files for each genome used in this study, can be found online in File S3 at https://github.com/megancamilla/Transposon-Mediated-transfer-of-ToxA/tree/master/S3_REPET_Files. Inverted repeats and TIRs flanking the *ToxA* gene and surrounding DNA were identified in Geneious with the Dotplot (Self) viewing tool, which is based on the EMBOSS 6.5.7 tool dotmatcher (http://emboss.sourceforge.net/apps/release/6.5/emboss/apps/dotmatcher.html) (96). The specific settings required to reproduce the line plot shown in Fig. 1 are as follows: Reverse complement=yes, Score Matrix=exact, window size = 100, Threshold = 75, and Tile Size = 1000.

### Whole-chromosome alignments.

Initial whole-genome alignments (WGA) were conducted using Lastz v1.02.00 or Mauve as implemented in Geneious v.9.1.8 (95–97). To obtain a clean gff or tab delimited file of high-identity segments, we developed mimeo, which parses the alignment output of LASTZ (97–99). mimeo and a description of its full features can found at https://github.com/Adamtaranto/mimeo. WGA of *ToxA^+^* and *toxa^−^* isolates was performed with LASTZ as implemented in mimeo with the following settings: mimeo-map --minIdt 60 --minLen 100 --maxtandem 40 --writeTRF. Candidate regions of transfer between the three species were also identified by WGA performed with LASTZ as described above using mimeo-map --minIdt 70 --minLen 100. All alignments were inspected manually in Geneious v9.1.8. Chromosomal alignments were plotted in R v3.5.2 using the genoPlotR package (100). The corrected and trimmed nanopore reads generated by Canu were used for realignment to the *de novo* assemblies. These reads were mapped to the assembly with Minimap2 with the following settings: minimap2 -x map-ont -a. The output pairwise alignment file (paf) was modified in R for plotting. The complete R_markdown document, which includes all of the code needed to reproduce Fig. 2, 3B, and 4, can be found online in File S4 at https://github.com/megancamilla/Transposon-Mediated-transfer-of-ToxA/tree/master/S4_Fig_Rmkds.

### BLAST searches.

All open reading frames identified within ToxhAT in isolate CS10 were translated using Geneious v9.18. These amino acid sequences were used as queries in tBLASTn searches on the NCBInr database and at JGI MycoCosm with the following settings: Blosum62 Matrix, Gap Costs: Existence 11 extension 1, e-value max 1e-10, max hits = 500 (last accessed 10 November 2018) (https://genome.jgi.doe.gov/programs/fungi/index.jsf) (101).

### Data availability.

Individual isolate accession numbers are listed in File S1 at https://github.com/megancamilla/Transposon-Mediated-transfer-of-ToxA/tree/master/S1_GenomeStats. All raw sequencing data are available under NCBI BioProject PRJNA505097. The P. nodorum SN15 Whole Genome Shotgun project has been deposited at DDBJ/ENA/GenBank under accession number SSHU00000000. Version SSHU01000000 is described in this paper. The P. nodorum Sn79-1087 Whole Genome Shotgun project has been deposited under accession numbers CP039668 to CP039689. The Whole Genome shotgun project and accession numbers for B. sorokiniana isolates are as follows: for CS10, SRZH00000000; for CS27, SRZG00000000; for WAI2406, SRZF00000000; for WAI2411, SRZE00000000. Transposon annotations and CS10 and CS27 gene annotations are available at https://github.com/megancamilla/Transposon-Mediated-transfer-of-ToxA.
