# Supplementary figures and images for: Transposon-Mediated Horizontal Transfer of the Host-Specific Virulence Protein ToxA between Three Fungal Wheat Pathogens
Source: mBio. 2019 Sep 10;10(5):e01515-19. doi: 10.1128/mBio.01515-19 (PMC6737239; doi:10.1128/mBio.01515-19)

# ToxhAT, ~14 kb

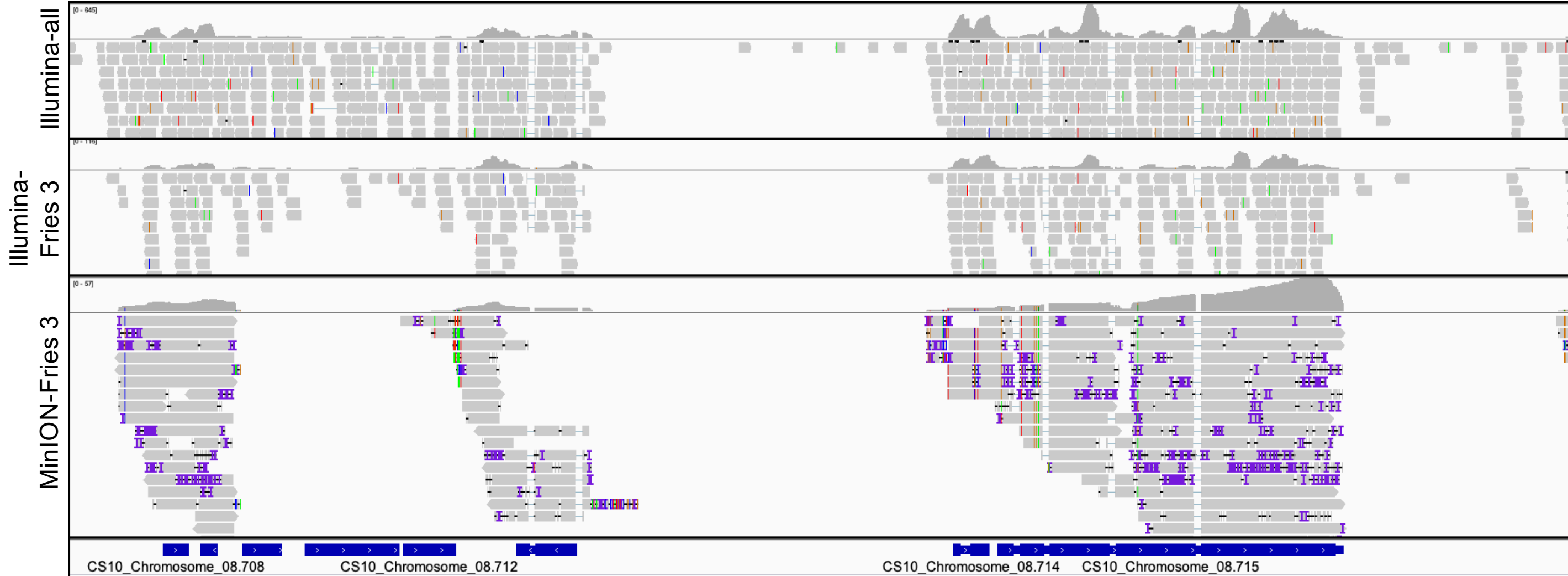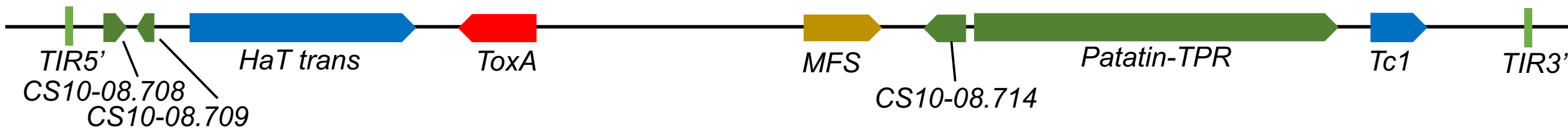

Supplement: FIG S1 [file mBio.01515-19-sf001.pdf]

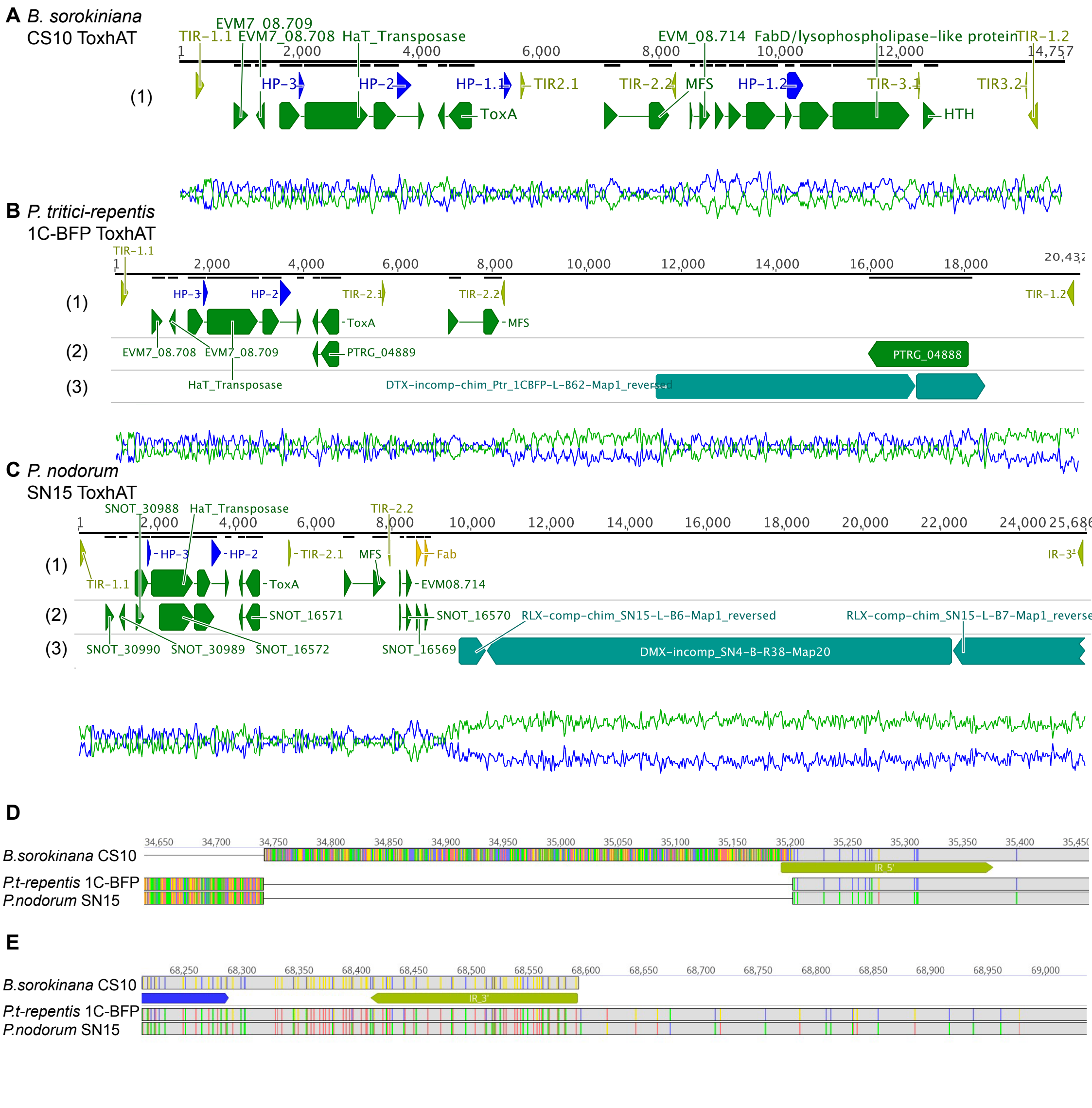

Supplement: FIG S2 [file mBio.01515-19-sf002.pdf]

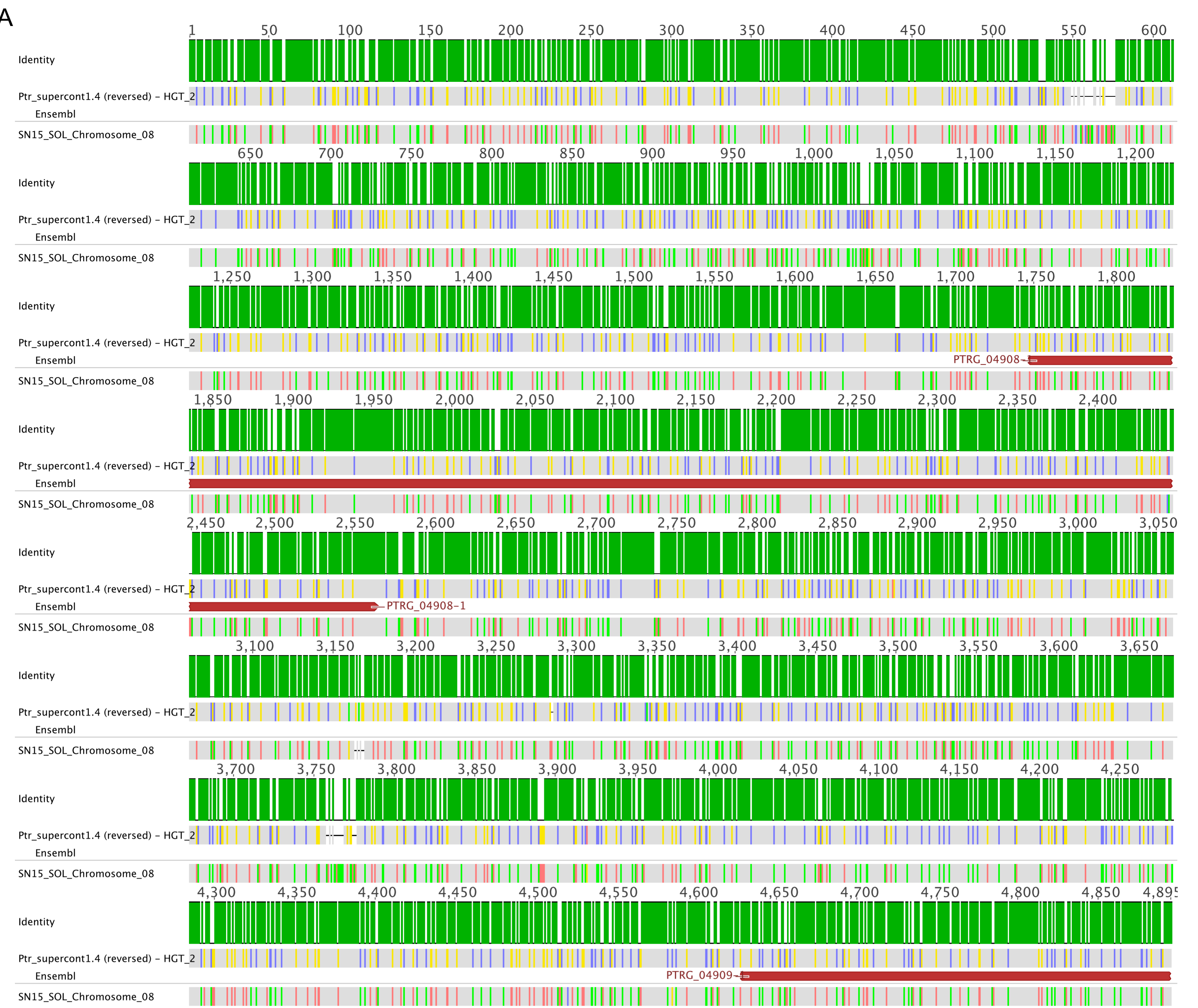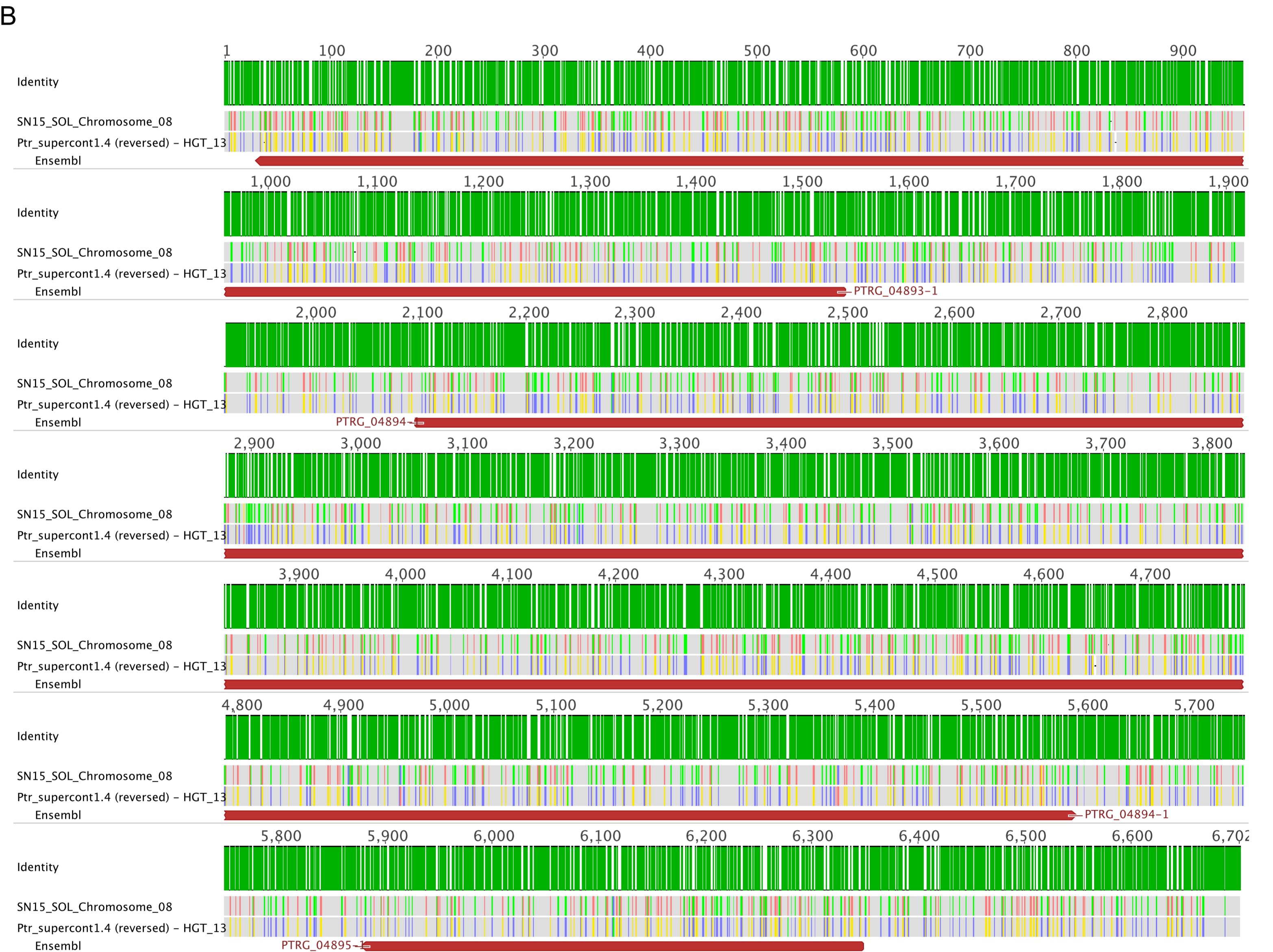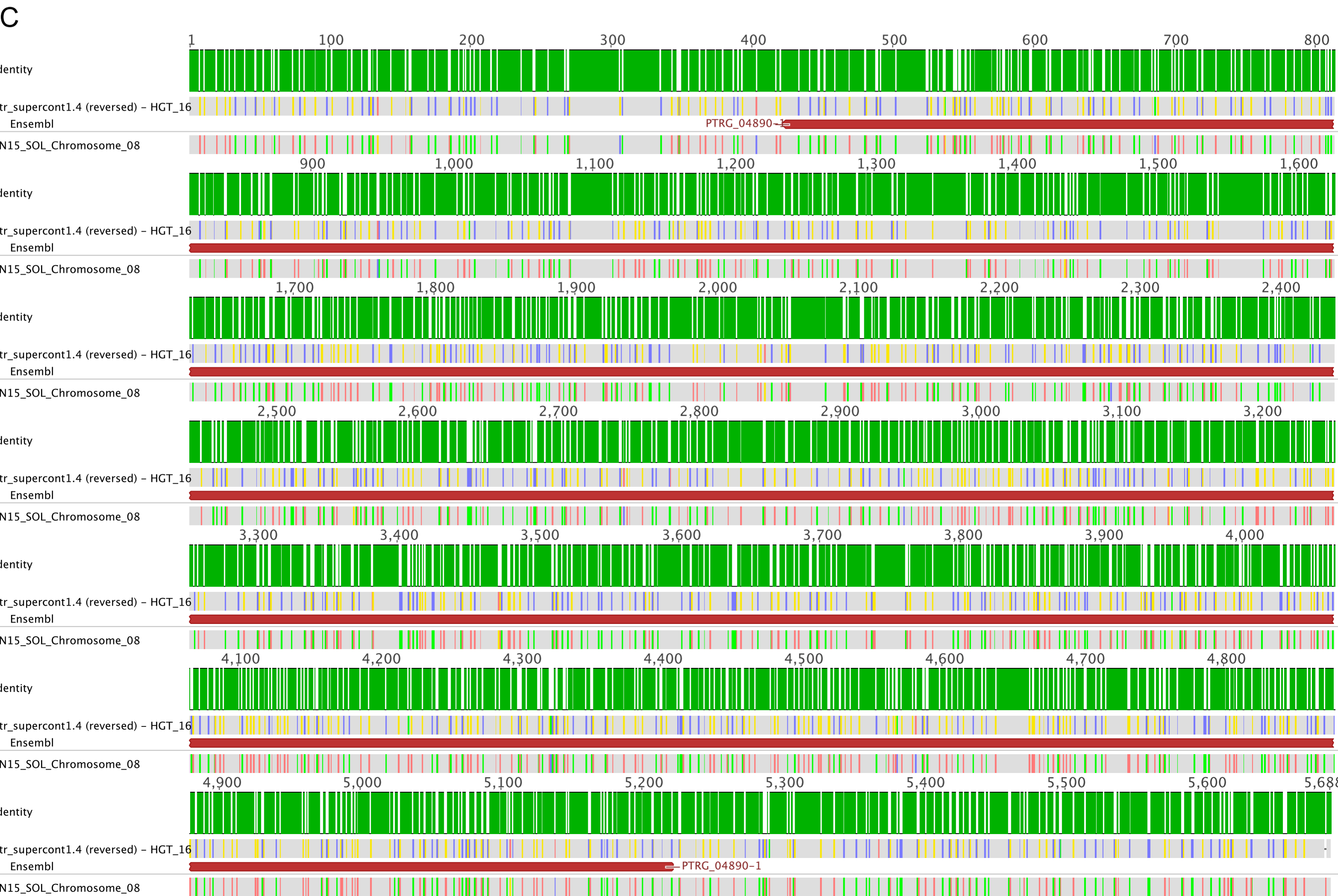

Supplement: FIG S3 [file mBio.01515-19-sf003.pdf]

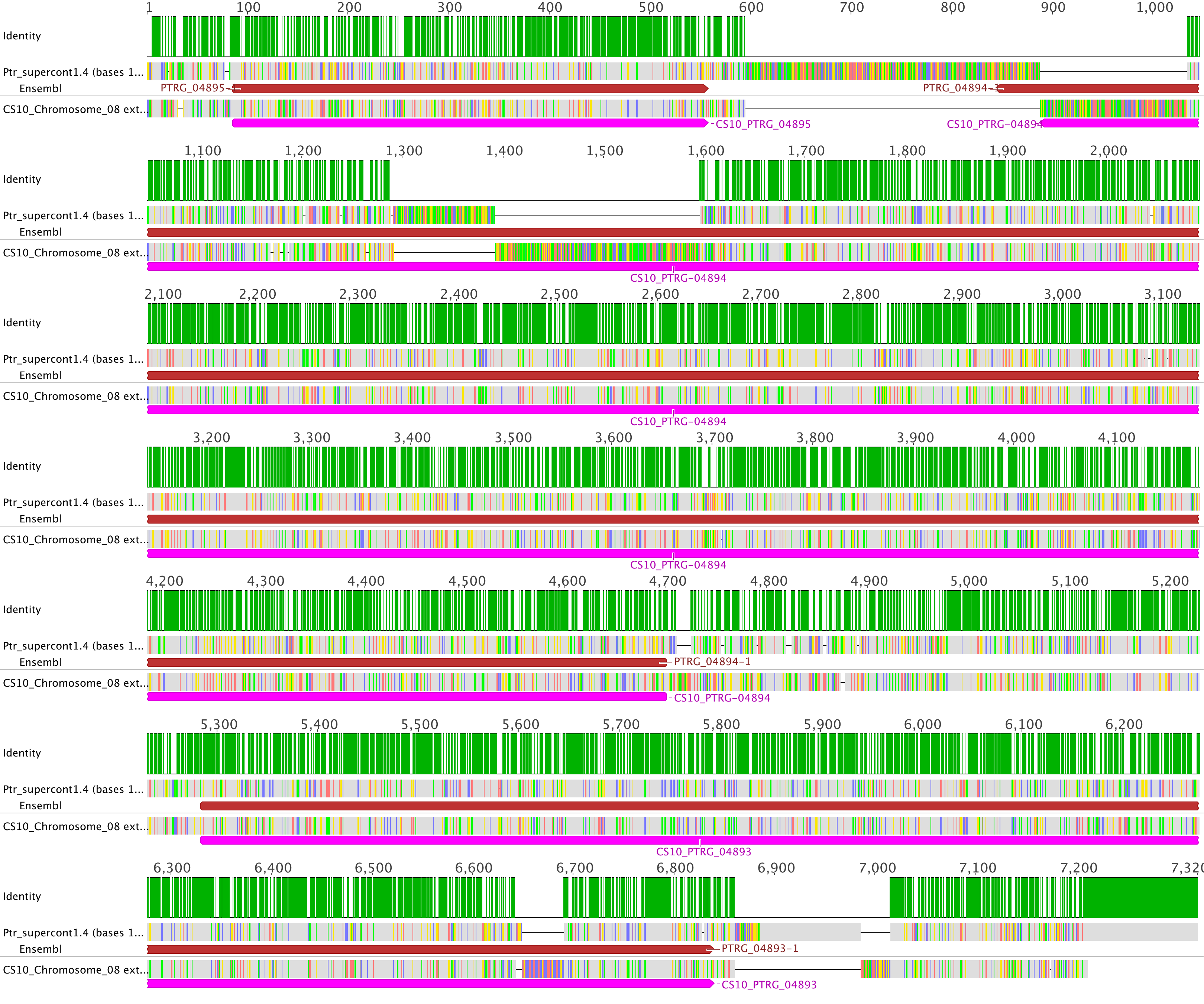

Supplement: FIG S4 [file mBio.01515-19-sf004.pdf]

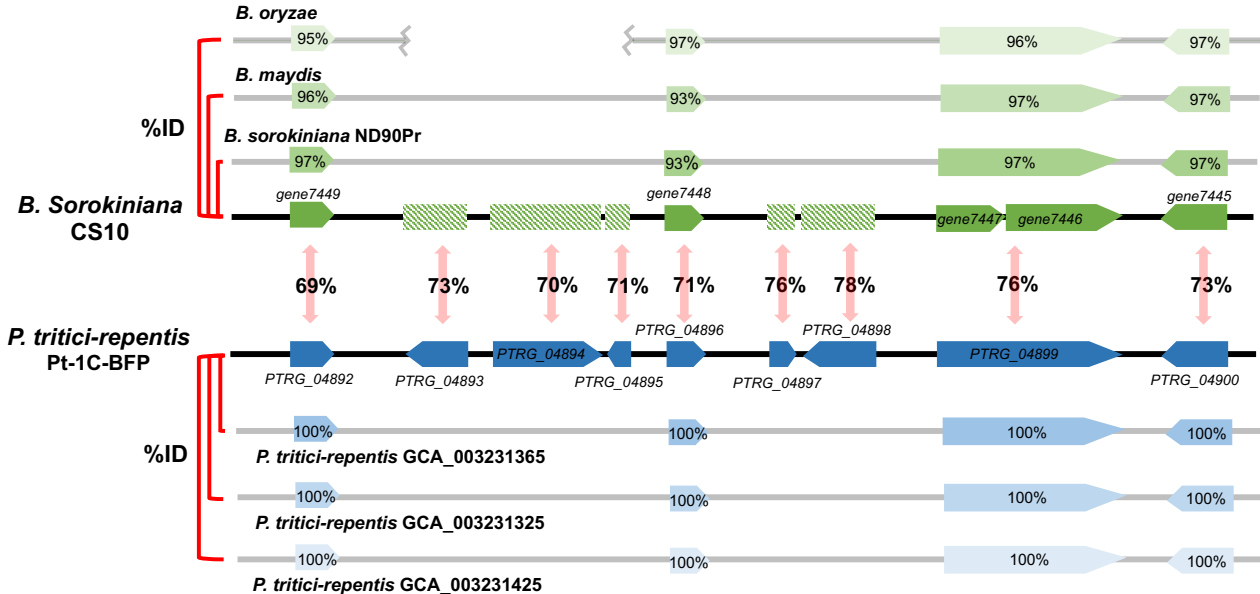

Supplement: FIG S5 [file mBio.01515-19-sf005.pdf]

A

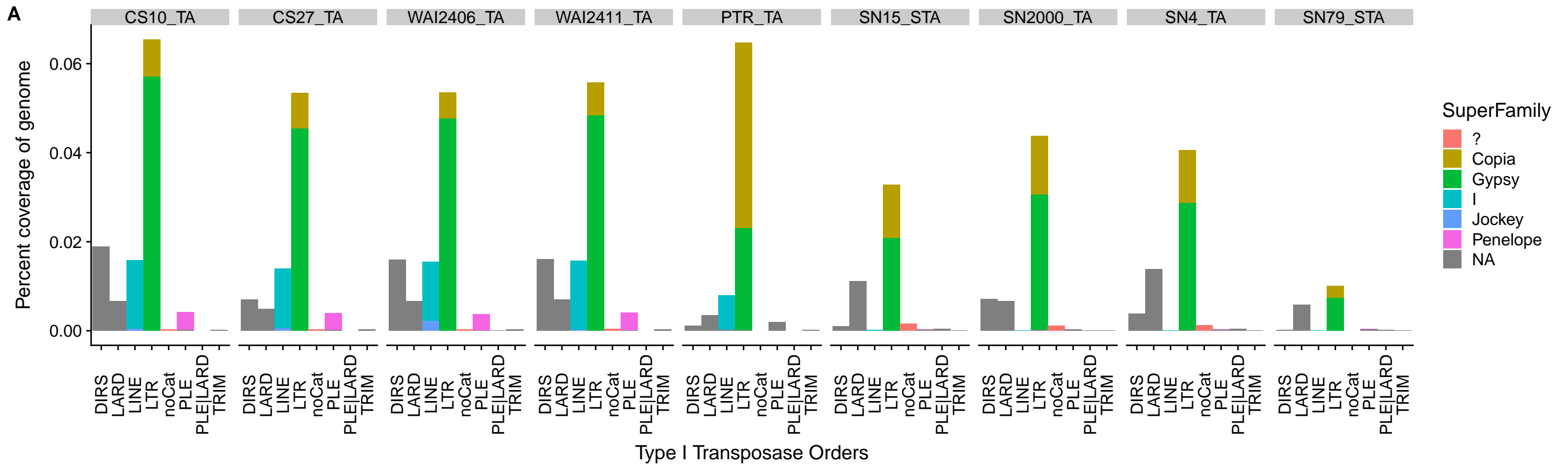

B

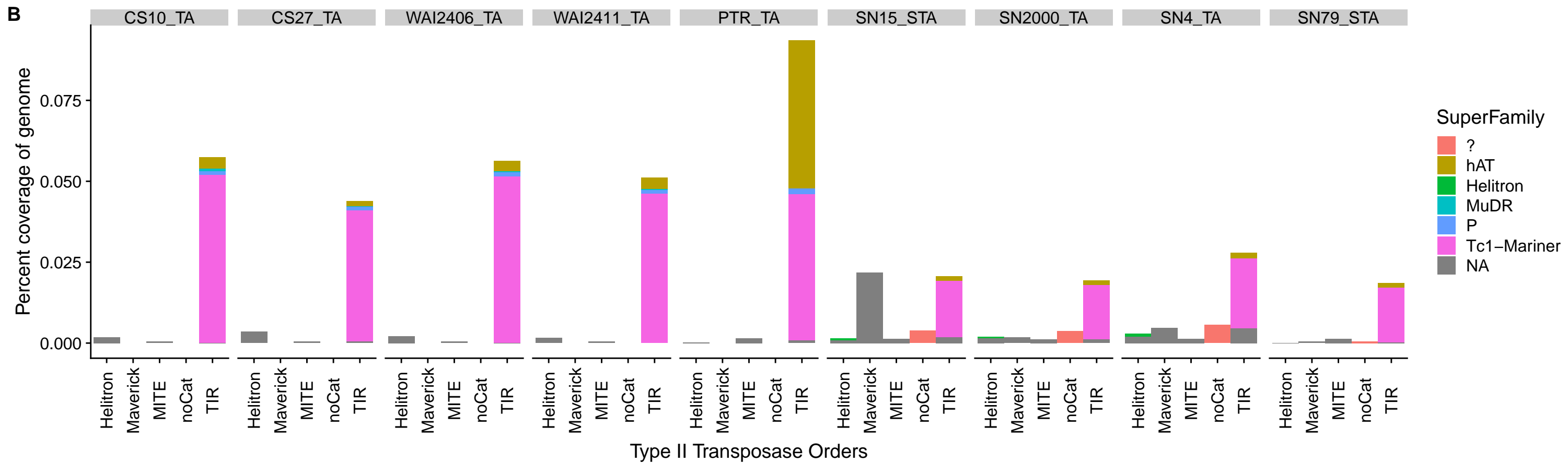

Supplement: FIG S6 [file mBio.01515-19-sf006.pdf]

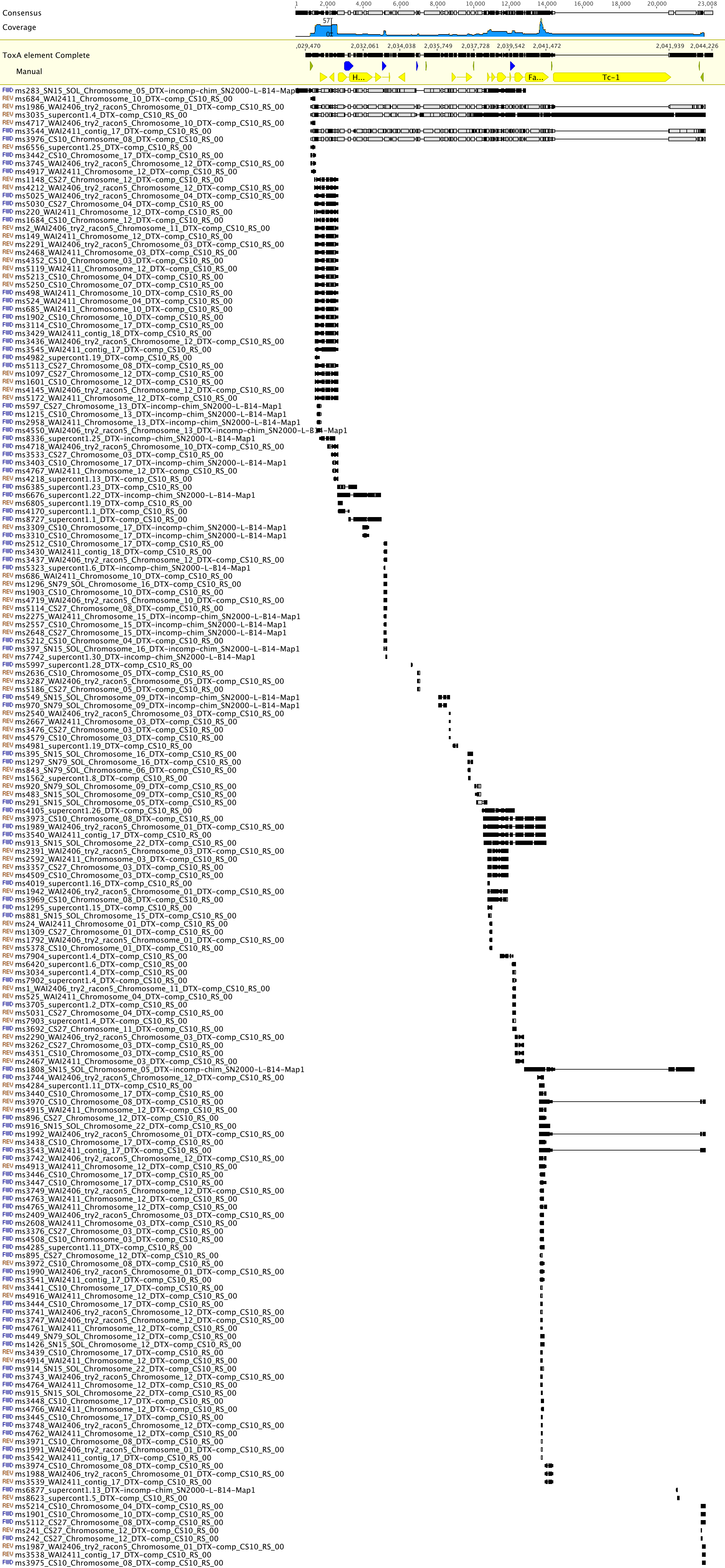

Supplement: FIG S7 [file mBio.01515-19-sf007.pdf]

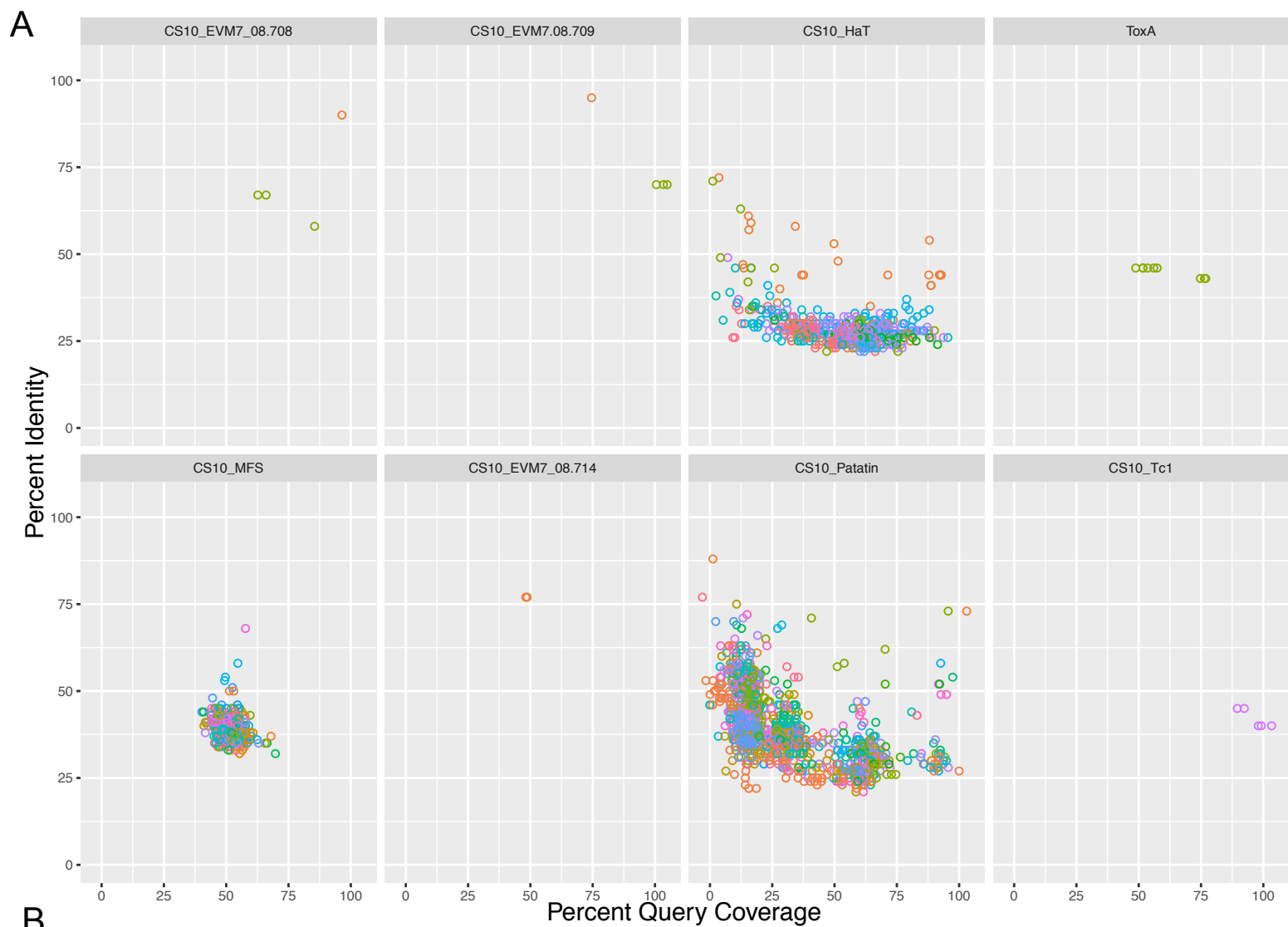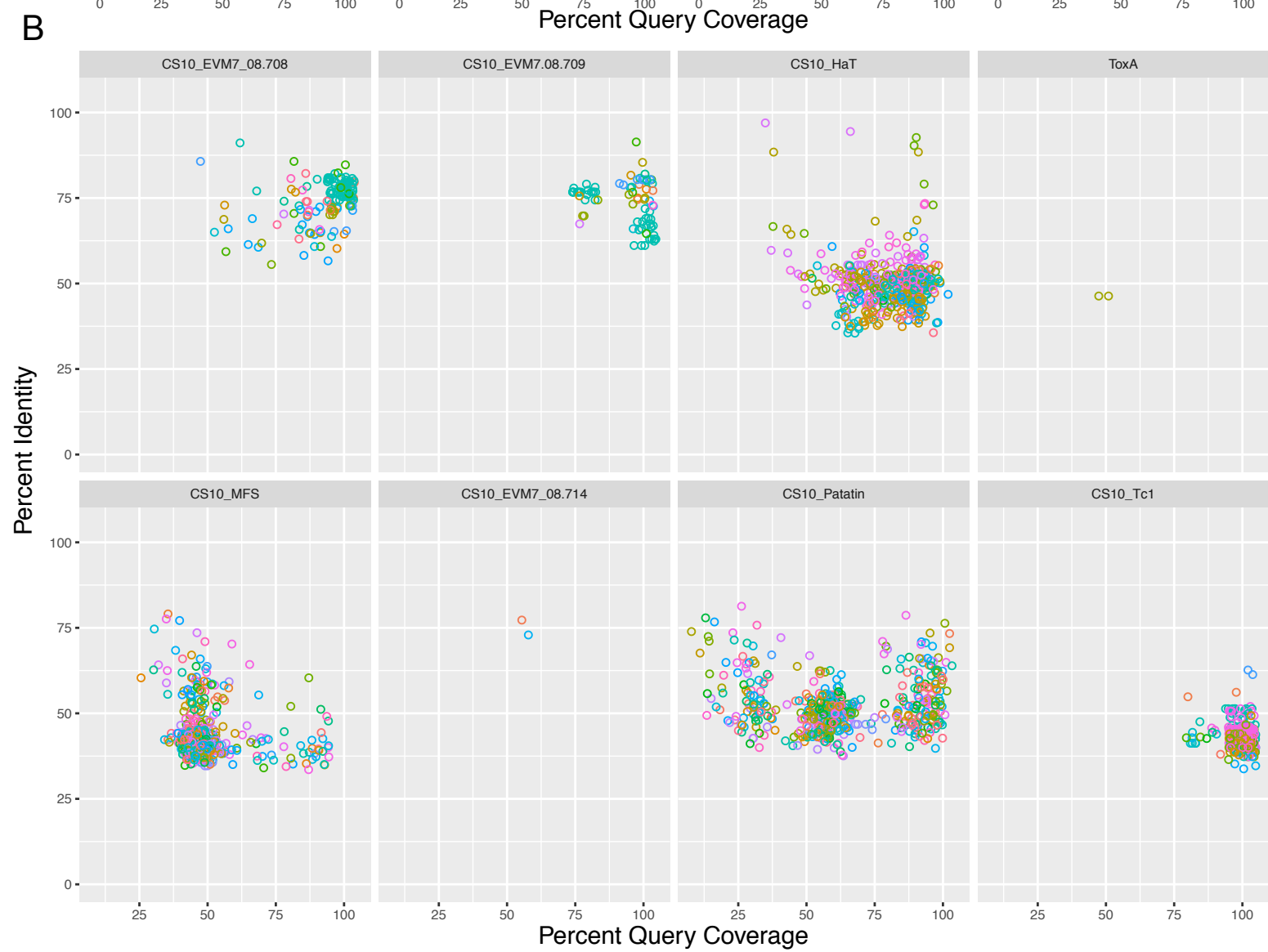

Supplement: FIG S8 [file mBio.01515-19-sf008.pdf]

A

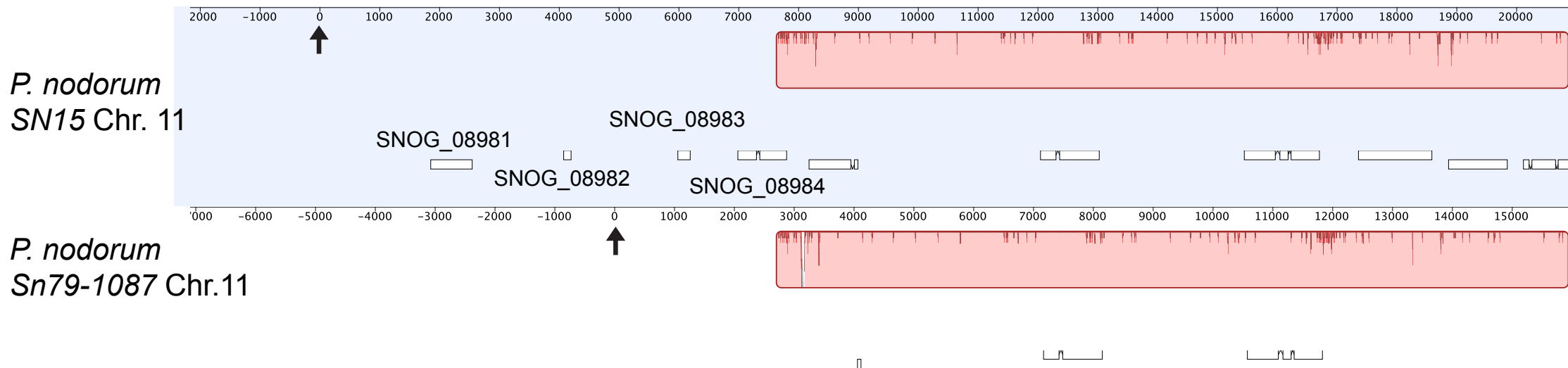

B

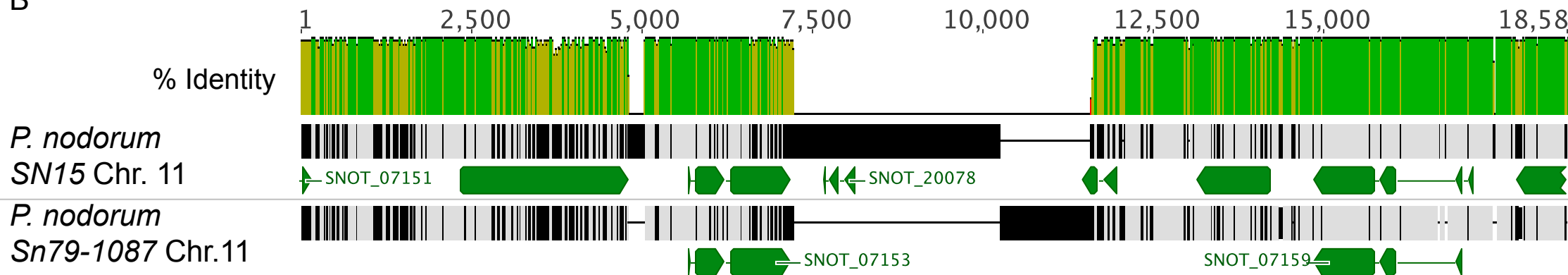

Supplement: FIG S9 [file mBio.01515-19-sf009.pdf]

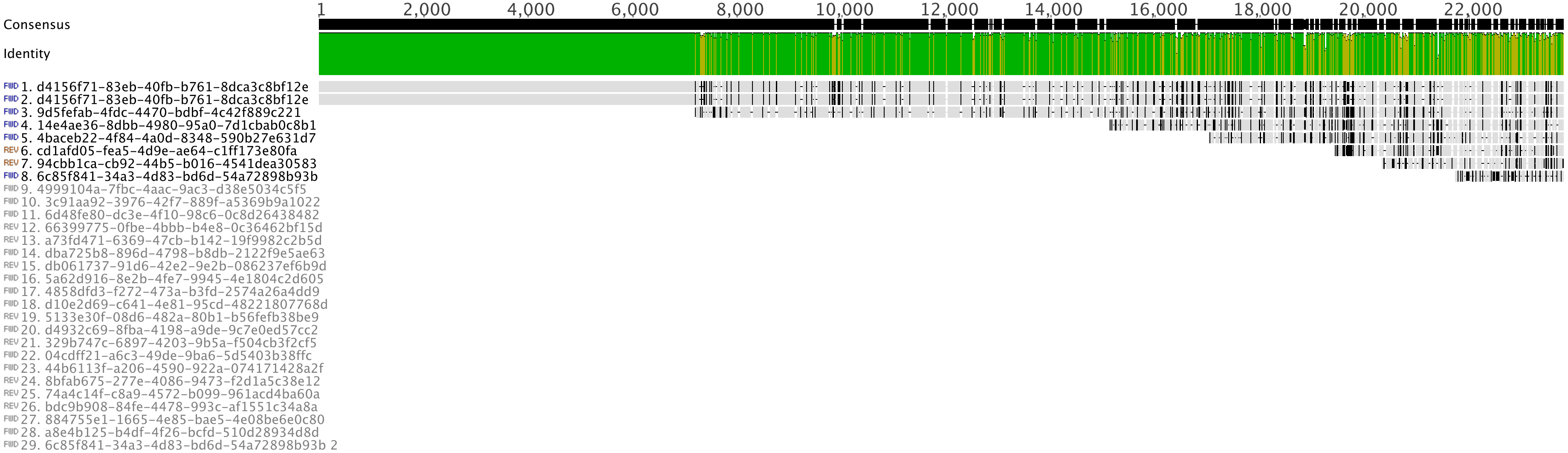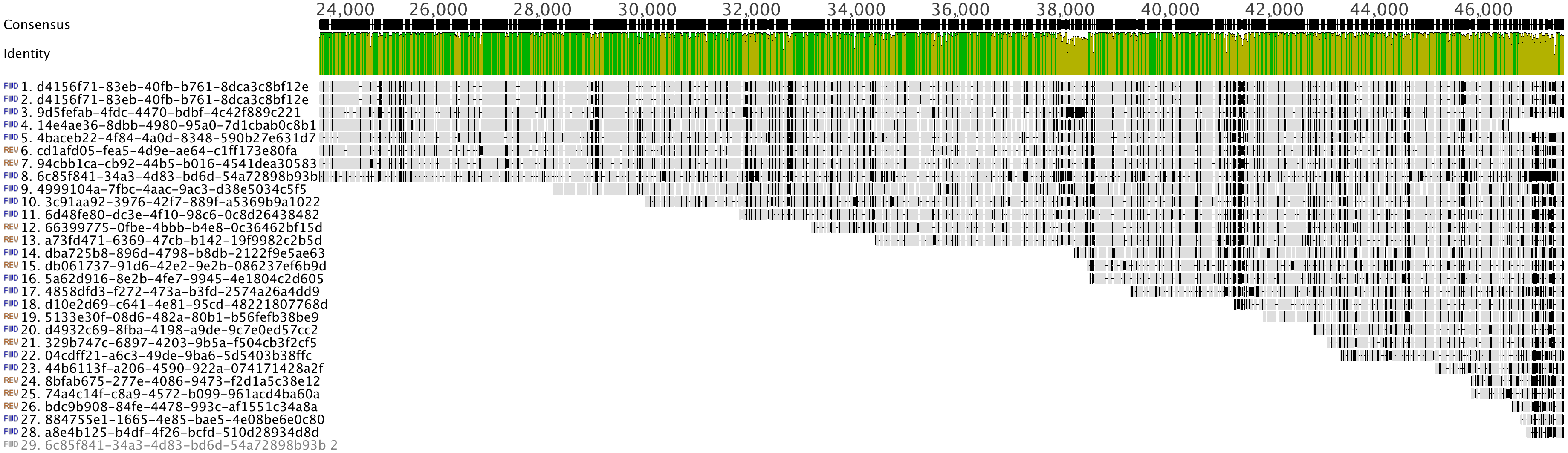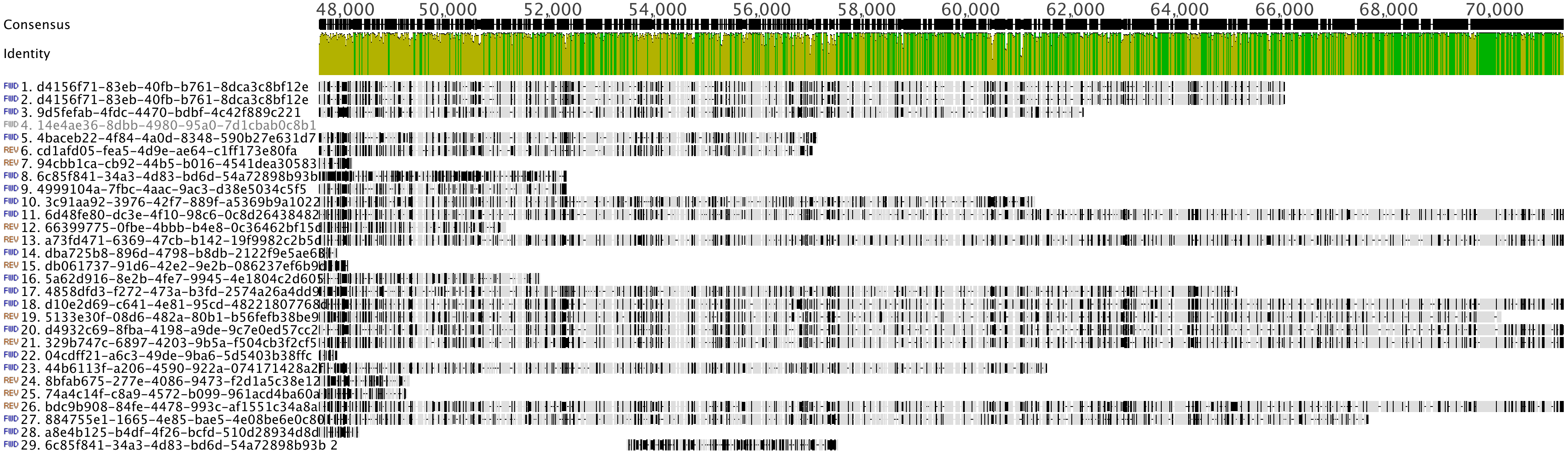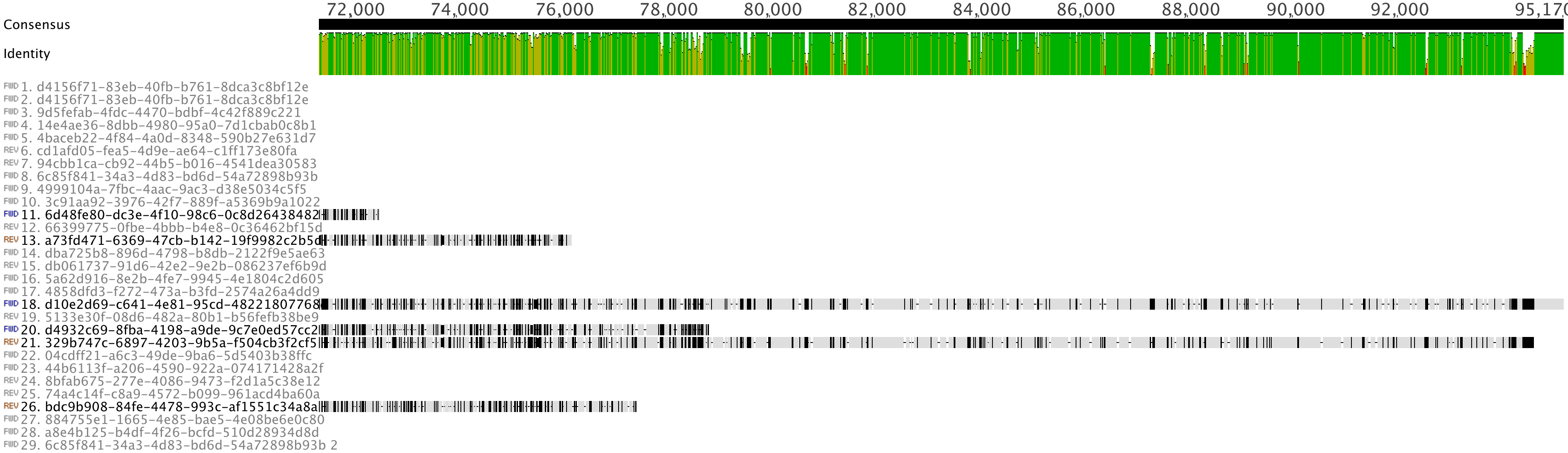

Supplement: FIG S10 [file mBio.01515-19-sf010.pdf]
